# The SARS-Coronavirus-Host Interactome: Identification of Cyclophilins as Target for Pan-Coronavirus Inhibitors

**DOI:** 10.1371/journal.ppat.1002331

**Published:** 2011-10-27

**Authors:** Susanne Pfefferle, Julia Schöpf, Manfred Kögl, Caroline C. Friedel, Marcel A. Müller, Javier Carbajo-Lozoya, Thorsten Stellberger, Ekatarina von Dall’Armi, Petra Herzog, Stefan Kallies, Daniela Niemeyer, Vanessa Ditt, Thomas Kuri, Roland Züst, Ksenia Pumpor, Rolf Hilgenfeld, Frank Schwarz, Ralf Zimmer, Imke Steffen, Friedemann Weber, Volker Thiel, Georg Herrler, Heinz-Jürgen Thiel, Christel Schwegmann-Weßels, Stefan Pöhlmann, Jürgen Haas, Christian Drosten, Albrecht von Brunn

**Affiliations:** 1 Bernhard-Nocht-Institute, Hamburg, Germany; 2 Institute of Virology, University of Bonn, Bonn, Germany; 3 Max-von-Pettenkofer Institute, Ludwig-Maximilians-University (LMU) Munich, München, Germany; 4 DKFZ, Heidelberg, Germany; 5 Institute for Informatics, LMU Munich, München, Germany; 6 Institute of Pharmacy and Molecular Biotechnology, Heidelberg University, Heidelberg, Germany; 7 IMMH, Albert-Ludwigs-University-Freiburg, Freiburg, Germany; 8 Institute of Immunobiology, Kantonsspital St. Gallen, Switzerland; 9 Institute of Biochemistry, University of Luebeck, Luebeck, Germany; 10 Institute of Virology, Hannover Medical School, Hannover, Germany; 11 Institute of Virology, Philipps-Universität Marburg, Marburg, Germany; 12 Institute of Virology, Tierärztliche Hochschule Hannover, Hannover, Germany; 13 Institute for Virology, Fachbereich Veterinärmedizin, Justus-Liebig Universität Gießen, Giessen, Germany; 14 Division of Pathway Medicine, University of Edinburgh, Edinburgh, United Kingdom; Vanderbilt University, United States of America

## Abstract

Coronaviruses (CoVs) are important human and animal pathogens that induce fatal respiratory, gastrointestinal and neurological disease. The outbreak of the severe acute respiratory syndrome (SARS) in 2002/2003 has demonstrated human vulnerability to (Coronavirus) CoV epidemics. Neither vaccines nor therapeutics are available against human and animal CoVs. Knowledge of host cell proteins that take part in pivotal virus-host interactions could define broad-spectrum antiviral targets. In this study, we used a systems biology approach employing a genome-wide yeast-two hybrid interaction screen to identify immunopilins (PPIA, PPIB, PPIH, PPIG, FKBP1A, FKBP1B) as interaction partners of the CoV non-structural protein 1 (Nsp1). These molecules modulate the Calcineurin/NFAT pathway that plays an important role in immune cell activation. Overexpression of NSP1 and infection with live SARS-CoV strongly increased signalling through the Calcineurin/NFAT pathway and enhanced the induction of interleukin 2, compatible with late-stage immunopathogenicity and long-term cytokine dysregulation as observed in severe SARS cases. Conversely, inhibition of cyclophilins by cyclosporine A (CspA) blocked the replication of CoVs of all genera, including SARS-CoV, human CoV-229E and -NL-63, feline CoV, as well as avian infectious bronchitis virus. Non-immunosuppressive derivatives of CspA might serve as broad-range CoV inhibitors applicable against emerging CoVs as well as ubiquitous pathogens of humans and livestock.

## Author Summary

Broad-range anti-infective drugs are well known against bacteria, fungi, and parasites. These pathogens maintain their own metabolism distinctive from that of the host. Broad-range drugs can be obtained by targeting elements that several of these organisms have in common. In contrast, target overlap between different viruses is minimal. The replication of viruses is highly interweaved with the metabolism of the host cell. A high potential in the development of antivirals with broad activity might therefore reside in the identification of host factors elemental to virus replication. In this work we followed a systems biology approach, screening for interactions between virus and host proteins by employing an automated yeast-two-hybrid setup. Upon binding of a viral protein to cyclophilins the screen led to the identification of the Calcineurin/NFAT pathway possibly being involved in the pathogenesis of SARS-Coronavirus. Secondly, cyclophilins were suggested to play an elemental role in virus replication since cyclosporin A inhibited replication of all Coronavirus prototype members tested. This large range of viruses includes common cold viruses, the SARS agent, as well as a range of animal viruses. For the first time this work shows that an undirected, systems-biology approach could identify a host-encoded, broad-range antiviral target.

## Introduction

Five distinct CoVs (SARS-CoV, hCoV-NL63, hCoV-HKU-1, hCoV-OC43, hCoV-229E) cause respiratory tract illness in humans, ranging from mild common cold to deadly virus-associated pneumonia [1]. At least seven different animal CoVs cause economically significant epizootics in livestock, and deadly disease in companion animals [1]. The agent of SARS was a novel CoV introduced into the human population from an animal reservoir, resulting in a highly lethal epidemic in 2002/2003 [1], [2]. A tremendous diversity of CoVs exists in complex mammalian and avian reservoirs [1], [3], [4]. Host switching is a common feature in CoV evolution, and novel epidemic CoV can emerge anytime [1], [3], [5]. Because the large diversity of CoVs complicates the design of vaccines, the identification of broad-range anti-CoV drug targets might indicate alternative approaches against CoV epidemics [1]. Broad range anti-CoV drugs would also be desirable to treat severe infections caused by known human and animal CoVs.

The SARS-CoV genome is predicted to encode 14 functional open reading frames, leading to the expression of up to 29 structural and non-structural protein products [1]. The functions of many of these proteins are poorly understood or unknown. To study the interplay of viral proteins with the host cell and to identify new targets involved in viral replication we have performed a genome-wide analysis of protein - protein interactions between the SARS-CoV and human proteins via a High-Throughput Yeast Two Hybrid Screen (HTY2H) [6], [7]. Within this framework we identified redundant interactions between SARS-CoV non-structural protein Nsp1 and a group of host proteins with peptidyl-prolyl cis-trans-isomerase activity, including the cyclophilins/immunophilins PPIA, PPIG, PPIH and FKBP1A, FKBP1B. These modulate the Calcineurin/NFAT pathway that plays an important role in immune cell activation [8], [9]. The NFAT family of transcription factors encodes four calcium-regulated proteins of which three (NFAT1, -2, -3) are expressed in a variety of cell types including T-cells, B-cells, mast cells, natural killer cells and eosinophils [8], [9]. NFAT activation regulates pivotal immune processes like apoptosis, anergy, and T-cell development. An essential activation step for NFAT is its dephosphorylation by the phospatase calcineurin A (CnA), resulting in the translocation of NFAT into the nucleus. Cyclosporin A (CspA) forms complexes with cyclophilins that bind to CnA, preventing its activity. This effect is used in transplant patients to prevent organ rejection by suppression of the immune system. Here we show that SARS-CoV nonstructural protein Nsp1, as well as full replicating SARS-CoV, enhance the CnA/NFAT pathway and induce NFAT-responsive promoters. Because interactions with upstream elements of the pathway were redundantly identified in a hypothesis-free virus-host interaction screen, the pathway is likely to play a significant role for virus replication. Indeed, an extensive panel of CoVs covering all three relevant virus genera was strongly inhibited by manipulation of cyclophilins using CspA.

## Results

### Interaction screening of the SARS-CoV ORFeome and host proteins

**Figure 1:**
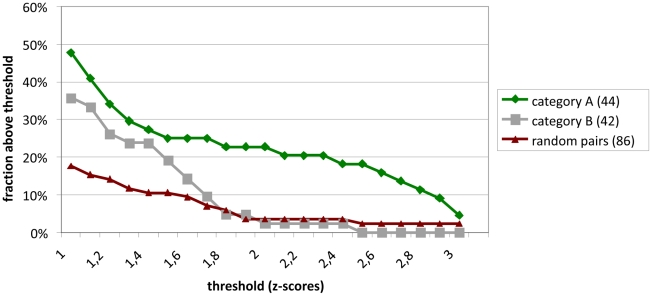
Validation of interactions detected by Y2H hybrid screening in LUMIER assays. Z-scores were calculated as described from duplicate experiments for 86 interactions observed in Y2H screens. 44 of the reproducible and specific interactions (category A) were tested. In addition, 42 interactions which were observed only once in a screen were tested (category B). These are compared to a negative reference set of non-interacting proteins. Shown in the Y-axis is the fraction of protein pairs above a threshold value (X-axis). The SARS interactions depicted here are listed in Table S1.

All SARS-CoV ORFs and a number of subfragments lacking transmembrane regions were cloned into eukaryotic expression vectors. Using HTY2H, these were screened against a cDNA library of very high complexity (1.4×10^7^) derived from human brain, as well as an additional library of individually-cloned full-length ORFs encoding 5000 human proteins. Inserts from positive yeast clones were sequenced and compared against GenBank. BLAST searches on 2287 DNA sequences yielded 942 different human gene hits. These were divided into four confidence categories: category A (highly confident interaction partners found more than once in one or several screens), category B (single hits), category C (sticky preys interacting with several to many bait proteins) and category D (3′-UTR cDNA regions or inserts in reverse orientation coding for unnatural peptides). We found 132, 383, 245, and 282 hits in categories A – D, respectively. For validation, the cDNAs of 86 category A and category B interaction candidates were cloned in-frame with the *Renilla reniformis* luciferase and overexpressed in HEK 293 cells. SARS-CoV ORFs were cloned in-frame with N-terminal protein A domains and co-expressed in the same cells. Protein A-directed immunoprecipitates retained on IgG-coated magnetic beads were identified by measuring *in-vitro* Luciferase activity. About 48% of category A candidates and 36% of category B candidates were confirmed positive with a Z-score >1 (**Figure 1**, see Materials and Methods for definition), corresponding to previous observations [10]. A list of validated category A and B HTY2H interactor candidates is provided in **Table S1**.

**Figure 2:**
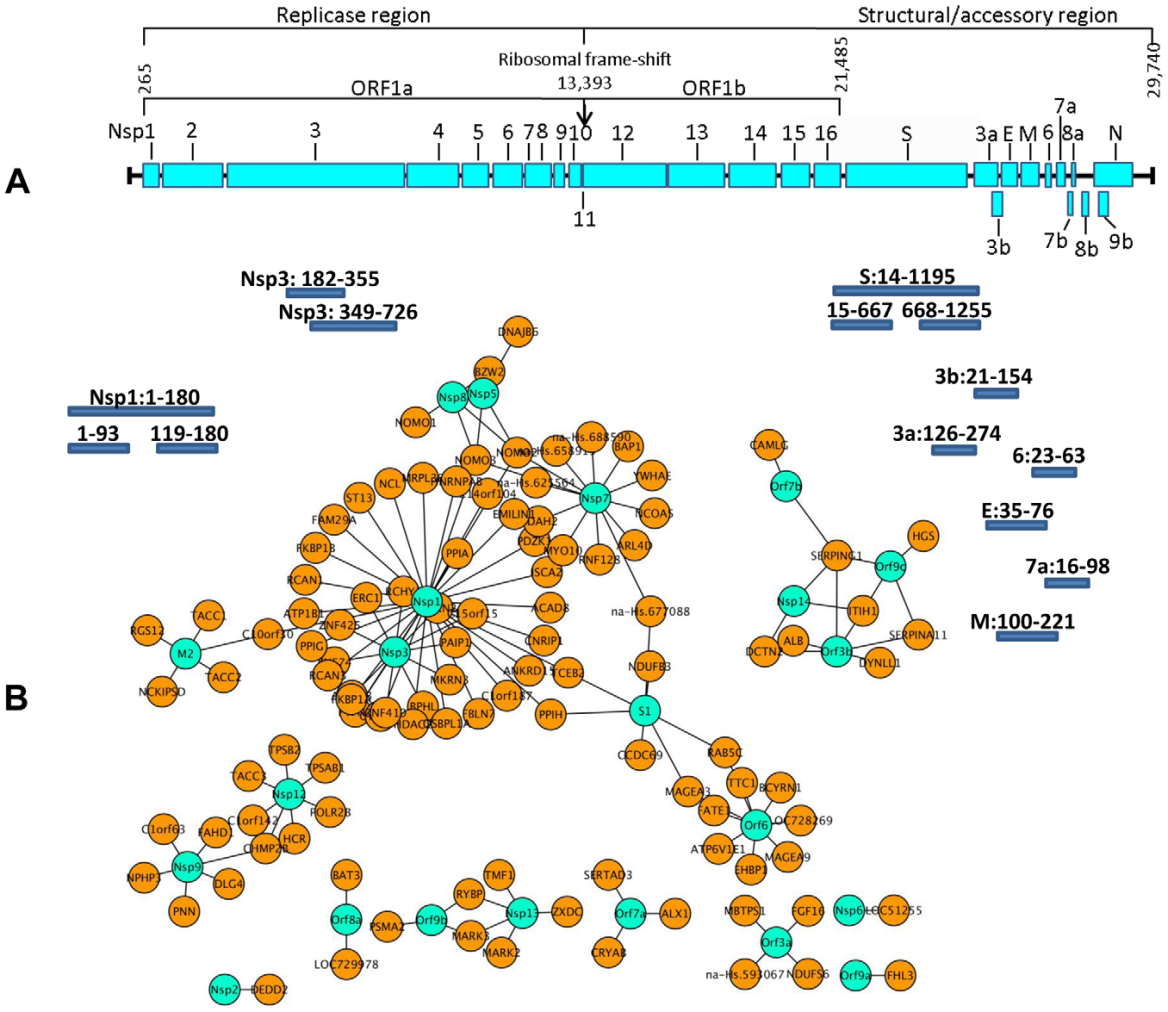
Localization of SARS-CoV ORFs and interaction network of virus host protein interactions. Figure **2A** shows an overview of the SARS-CoV ORFs used as the basis for the construction of the viral ORFeome [6]. Individual ORFs were PCR amplified by primers specific for the predicted N- and C- terminal ends including sequences of the GATEWAY cassette. Additionally, hydrophobic sequences were deleted from ORFs containing transmembrane regions. Amino acid positions of these fragments (small bars, not drawn to scale) are given behind the respective ORF name and refer to the starting position of each individual ORF. Hypothetical ORF14 [57] was also subcloned. Figure **2B** shows highly confident interaction partners of SARS-CoV ORF as identified by ORFeome-wide Y2H screen. Viral proteins are shown in turquoise, and are connected to direct cellular interaction partners shown in orange.

For an overall estimate of plausibility, more than 5,000 Medline abstracts mentioning “SARS” or “Coronavirus” were screened using the text mining program *syngrep*, scanning for the mentioning of human protein designations and synonyms. Abstracts mentioning YTH or co-immunoprecipitation assays were specifically sought. Twenty-eight CoV-/host protein interactions were identified in the literature, as listed in **Table S2**. It was then determined how these literature hits overlapped with the lists of candidate interactors as identified by HTY2H screening in different confidence levels. Using a hypergeometric test, the fractions of overlap were compared to the fraction of literature hits in the list of search terms (31,941 human proteins used for text-mining). Abstracts were enriched for proteins identified as SARS-CoV interaction partners both in the high confidence and the complete data sets (**Table S3 and Table S4**). **Figure 2** summarizes highly confident interactions identified in the overall screen and GO [11], [12] analysis. SARS-CoV proteins were found to preferentially target protein complex subunits (**Table S5 and Table S6**). Of 9 complexes which were targeted through ≥4 subunits, 4 complexes were found to be significantly enriched: The respiratory chain complex I (7 subunits targeted by SARS-CoV, p-value ∼0.036), the cytoplasmic ribosome (10 subunits targeted by SARS-CoV, p-value ∼0.036), in particular the 60S ribosomal subunit (7 subunits targeted by SARS-CoV, p-value ∼0.036) and the LCR-associated remodeling complex which is involved in DNA conformation modification (4 subunits targeted by SARS-CoV, p-value ∼ 0.039). Furthermore, the analysis of the centrality of SARS targets within the human interaction network (**Figure S1**) indicated that SARS-CoV proteins target both highly interactive proteins (hubs) as well as so-called bottleneck proteins which are central to many of the shortest paths in their networks [13] (**Figure S2**). **Table S4** summarizes GO results for SARS-CoV nonstructural protein Nsp1, a protein yielding particularly interesting candidate interaction networks. Interactions between Nsp1 and several members of the class of immunophilins (PPIA, PPIG, PPIH, FK506-binding proteins FKBP1A and -B) and calcipressins (RCAN1 and -3) were selected for experimental confirmation.

### The N-terminal part of SARS-CoV Nsp1 influences NFAT activation by interacting with several immunophilins and a calcipressin

**Figure 3:**
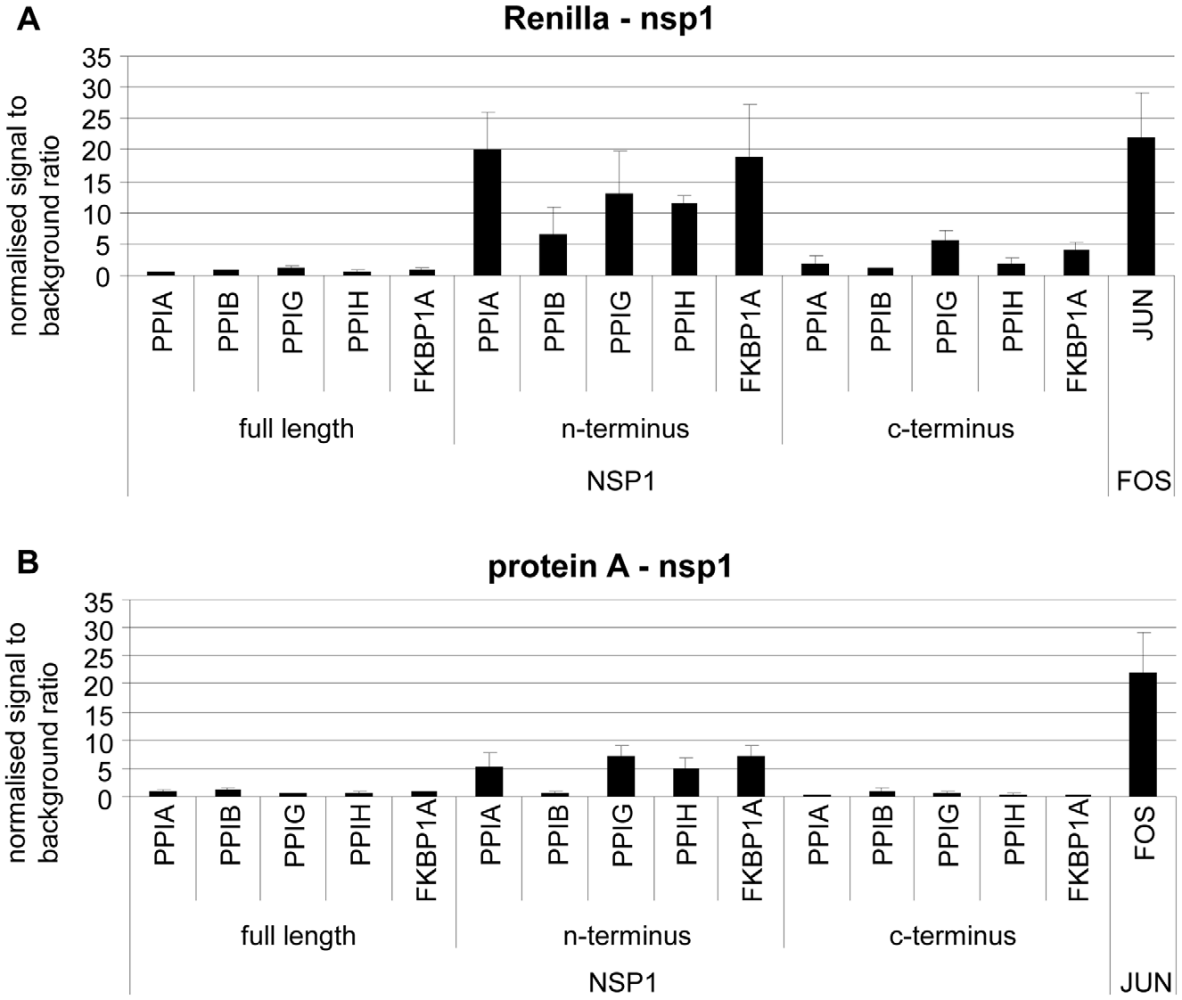
Validation of SARS-CoV Nsp1 interaction with immunophilins (cyclophilins PPIA, PPIB, PPIG, PPIH and FK506-binding protein FKBP1A) and calcipressin (RCAN3) by modified Lumier assay. Three versions of Nsp1 (Nsp1fl  =  aa 1–180, Nsp1N-terminus  =  aa 1–93 and Nsp1 C-terminus  =  aa119–180) and human cDNAs were cloned into protein A and *Renilla* Luciferase fusion vectors. *Renilla*-Nsp1 (**A**) or protein A-Nsp1 (**B**) was cotransfected with each respective cDNA into HEK293 cells. Complexes were purified via IgG-coated magnetic beads and Luciferase activity was determined as a measure for binding activity. As a positive control the very strongly interacting jun and fos genes were used. On the y-axis normalized signal to background ratios are shown.

**Figure 4:**
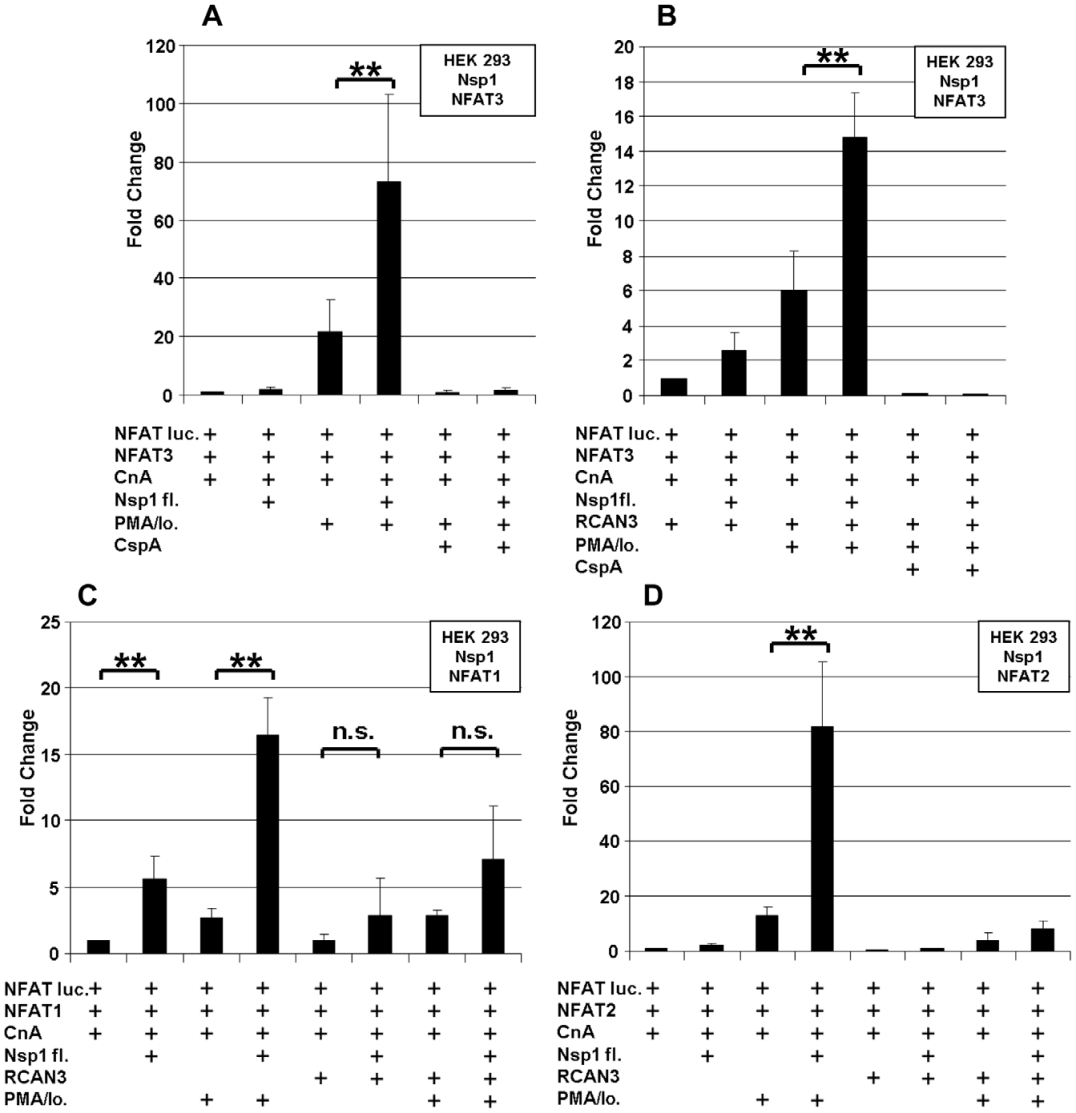
SARS-CoV Nsp1 full length (Nsp1fl) induces NFAT-regulated gene expression *in vitro* independently of the NFAT molecular species, and the calcipressin RCAN3 extenuates the effect. HEK293 cells were transiently cotransfected with NFAT reporter plasmid (NFAT luc) and expression plasmids encoding NFAT3, Calcineurin (CnA) and SARS-CoV Nsp1fl (**A**). RCAN3 was additionally expressed in (**B**). In (**C**) and (**D**), NFAT1 and NFAT2 species were expressed instead of NFAT3, respectively. The respective empty plasmid vector DNA was added to each individual transfection setup in order to obtain identical DNA concentrations. After transfection cells were cultured in absence or presence of the calcineurin stimulators PMA and ionomycin (PMA/Io.) and the NFAT-pathway inhibitor Cyclosporin A (CspA). ** *P*<0.01.

The immunophilin proteins (cyclophilins and FK506-binding proteins) are all known to bind to CnA in combination with inhibitory molecules, and to influence the CnA/NFAT pathway that plays a major role in the establishment of T-cell immune response [14]. For a more detailed mapping of HTY2H hits, PPIA, PPIB, PPIG, PPIH, FKBP1A and RCAN3, and three versions of Nsp1 [Nsp1(aa 1–180), Nsp1(aa 1–93) and Nsp1(aa 119–180)] were cloned into LUMIER assay vectors to yield luciferase and protein A fusion proteins, respectively. Although PPIB was not identified as an interactor by Y2H it was included in the experiment as it is known to bind to the HIV-1 gag protein [15] and to the HCV NSB5 protein [16]. All tested proteins interacted with Nsp1(aa 1–93), suggesting redundant interactions of SARS-CoV with the CnA/NFAT pathway via the N-terminal part of Nsp1 (**Figure 3**). To examine the functional consequences of Nsp1 expression on NFAT activity, NFAT and CnA cDNAs were overexpressed in HEK 293 cells. Parallel experiments in Jurkat cells were done without overexpression due to their constitutive activity of the CnA/NFAT pathway. The CnA/NFAT pathway was stimulated by addition of PMA (40 ng/ml) and ionomycin (2 µM) to the culture medium. In both cell lines treated this way, expression of Nsp1 did not induce NFAT activity directly, but increased significantly the stimulatory effect of PMA/ionomycin on NFAT activation (**Figure 4A**). The increase in NFAT activity could be blocked by CspA, an inhibitor of the NFAT pathway (**Figure 4A and B**). Coexpression of the calcipressin RCAN3 as shown in Figure 4B attenuated the overall stimulating effect on the NFAT activity. In contrast, coexpression of other CoV proteins or coexpression of PPIA, PPIH, FKBP1A did not impact NFAT activity (data not shown). Experiments up to this point employed overexpression of NFAT3. As different NFAT species are expressed depending on cell type [17], NFAT1 and NFAT2 were alternatively expressed and compared in the same assay. For both species essentially the same influence of Nsp1 on PMA/ionomycin-dependent stimulation was seen (**Figure 4C and D**). Altogether this suggested a broad effect of Nsp1 on NFAT activation that is mediated via the canonical NFAT activation pathway including CnA.

**Figure 5:**
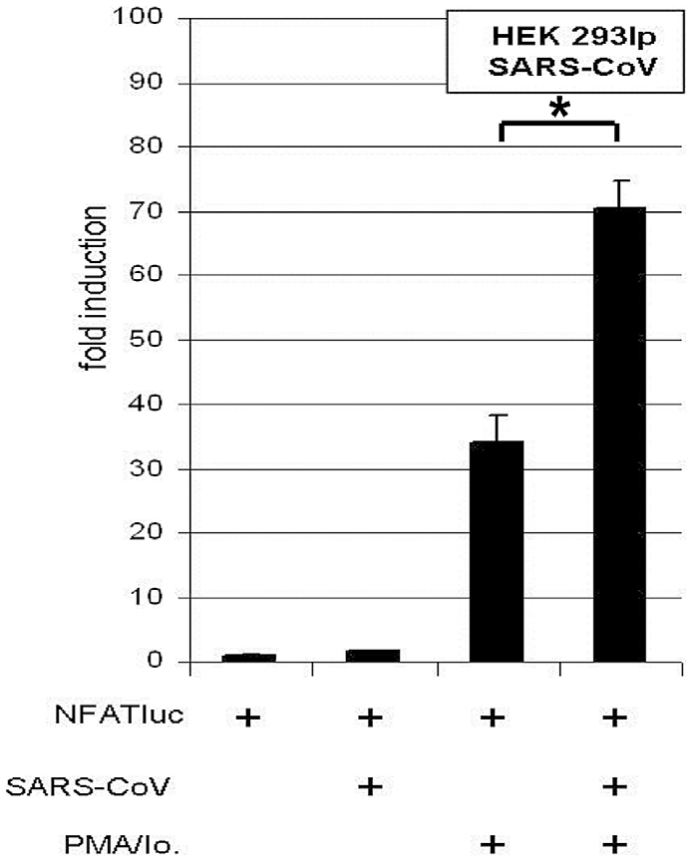
SARS-CoV isolate “Hongkong” induces NFAT-regulated gene expression. HEK 293lp cells were transiently transfected with NFAT reporter plasmid (NFATluc). 24 h post transfection cells were infected with SARS-CoV isolate “Hongkong” (SARS-CoV HK) and the medium was supplemented with the calcineurin stimulators PMA and ionomycin (PMA/Io.). 17 h post infection the luciferase readout was carried out. * *P*<0.05.

In order to determine the extent of PMA/ionomycin-dependent NFAT activation during virus infection, HEK 293 lp cells (lp = low passage) with a short passage history were infected with SARS-CoV at an MOI = 1. These cells had been previously demonstrated to support SARS-CoV replication, in contrast to common HEK 293 cells [18], [19]. **Figure 5** shows that the CnA/NFAT pathway was induced in the context of SARS-CoV infection at considerable extent, and in a PMA/ionomycin-dependent way.

### Effects on cytokine induction

**Figure 6:**
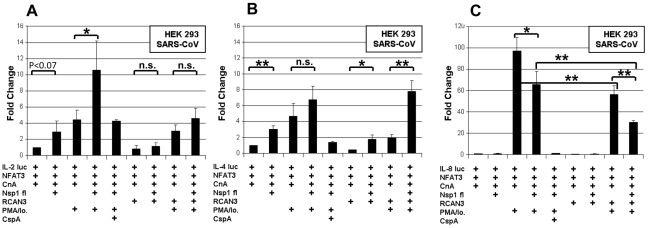
Influence of Nsp1 on Interleukin promoters. HEK293 cells were transiently cotransfected with interleukin reporter plasmids IL2 luc (**A**), IL4 luc (**B**), IL8 luc (**C**) and expression plasmids encoding NFAT3, CnA and either SARS-CoV Nsp1fl or the empty plasmid vector. All experiments were also done with an additional overexpression of the Calcipressin RCAN3. After transfection cells were cultured in absence or with the calcineurin stimulators PMA/Io. and the inhibitor CspA. * *P*<0.05; ** *P*<*0.01.*

Viruses may interfere with cytokine induction, but on the other hand, may also induce cytokine genes directly. To examine whether the Nsp1-mediated, PMA/ionomycin-dependent activation of NFAT may cause specific induction of relevant cytokines, HEK293 cells were co-transfected with the plasmids described above, except that the NFAT reporter plasmid was replaced by luciferase reporter plasmids carrying the IL-2, IL-4 and IL-8 promoters, respectively (**Figure 6**). Expression of Nsp1 induced the IL-2 promoter significantly by a factor of about 2.5 (**Figure 6A**). This effect was inhibited by CspA and RCAN-3, suggesting dependence on the CnA/NFAT pathway. The IL-4 promoter activity was not significantly elevated by Nsp1 expression in the presence of PMA/ionomycin (**Figure 6B**). Its activity was decreased in the presence of CspA but not RCAN-3. The IL-8 promoter was induced by PMA/ionomycin alone, but significantly downregulated by a factor of about 1.8 in additional presence of Nsp1 (**Figure 6C**). Expression of RCAN-3 reduced IL-8 promoter activity levels to about half, while CspA inhibited the promoter completely. In Jurkat cells, which express endogenous NFAT3 and CnA, the Nsp1 protein did not induce the IL-2 promoter. The slight induction of IL-4 and the downregulation of the IL-8 promoter activities in presence of Nsp1 (about twofold) were similar to effects seen in HEK 293 cells. These results suggested that Nsp1 expression had the strongest influence on the IL-2 promoter.

**Figure 7:**
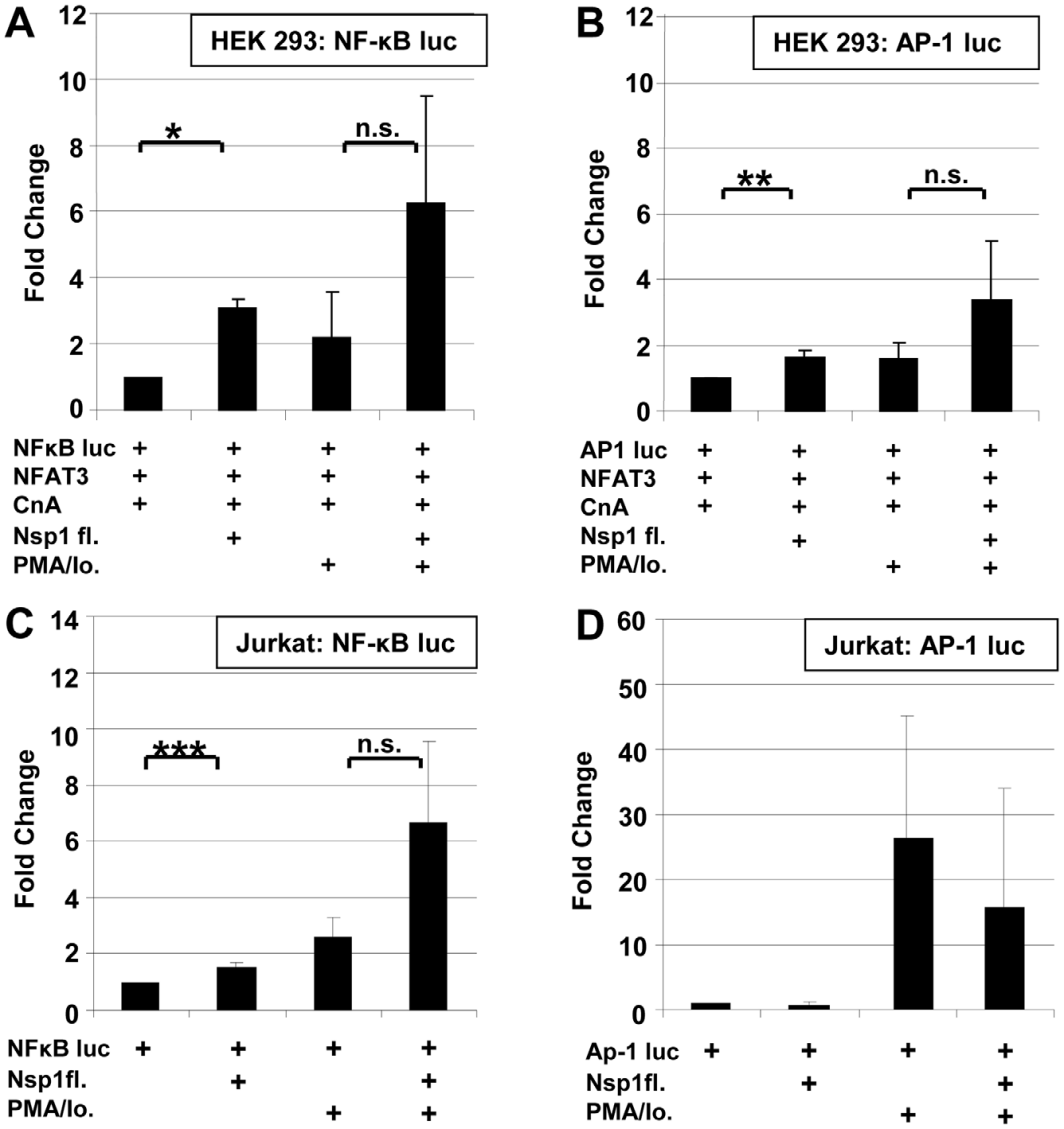
Influence SARS-CoV Nsp1fl on transcription factors NF-κB and AP-1. HEK 293 and Jurkat cells were transiently co-transfected with NFκB-luc (**A,C**) or AP-1luc (**B,D**) and SARS-CoV Nsp1fl or an empty vector. Induction of the cells was carried out with PMA/Io. * *P*<0.05; ** *P*<*0.01,* ***P<*0.005*.

Next to NFAT, transcription factors NFκB and Activating Protein 1 (AP-1) determine IL-2 regulation [20]. NFAT, AP-1 and NFκB binding sites are juxtaposed in the IL-2 promoter, and it has been shown that NFAT and AP-1 act in a cooperative manner on the promoter while NFkB has enhancing function [17]. Simultaneously, NFκB induces the IFN-beta gene by binding to the PRDII DNA element [21]. The latter is a more sensitive assay of NFκB nuclear translocation upon viral infection. To examine potential direct effects of Nsp1 on NFκB nuclear translocation and AP-1, HEK 293 and Jurkat cells were cotransfected with SARS-CoV Nsp1fl and p55A2luc containing repeated PRDII elements or pAP-1-luc containing the AP-1 binding site of the IL-2 promoter (**Figure 7**). Overexpression of Nsp1fl as well as treatment with PMA/ionomycin, respectively, caused small but significant luciferase increases in both cell lines. The combined expression of Nsp1fl with PMA/ionomycin treatment led to significant induction of PRDII by a factor of about 6 in both cell lines. The AP-1 promotor was only slightly upregulated in HEK293 and downregulated in Jurkat cells. This indicated a co-involvement of NFκB but not of AP-1 in the induction of IL-2 by Nsp1, suggesting dependence mainly on the NFAT pathway. In summary, SARS-CoV caused relevant and specific induction of IL-2 by activating the NFAT pathway via Nsp1.

### CspA inhibits replication of CoVs

**Figure 8:**
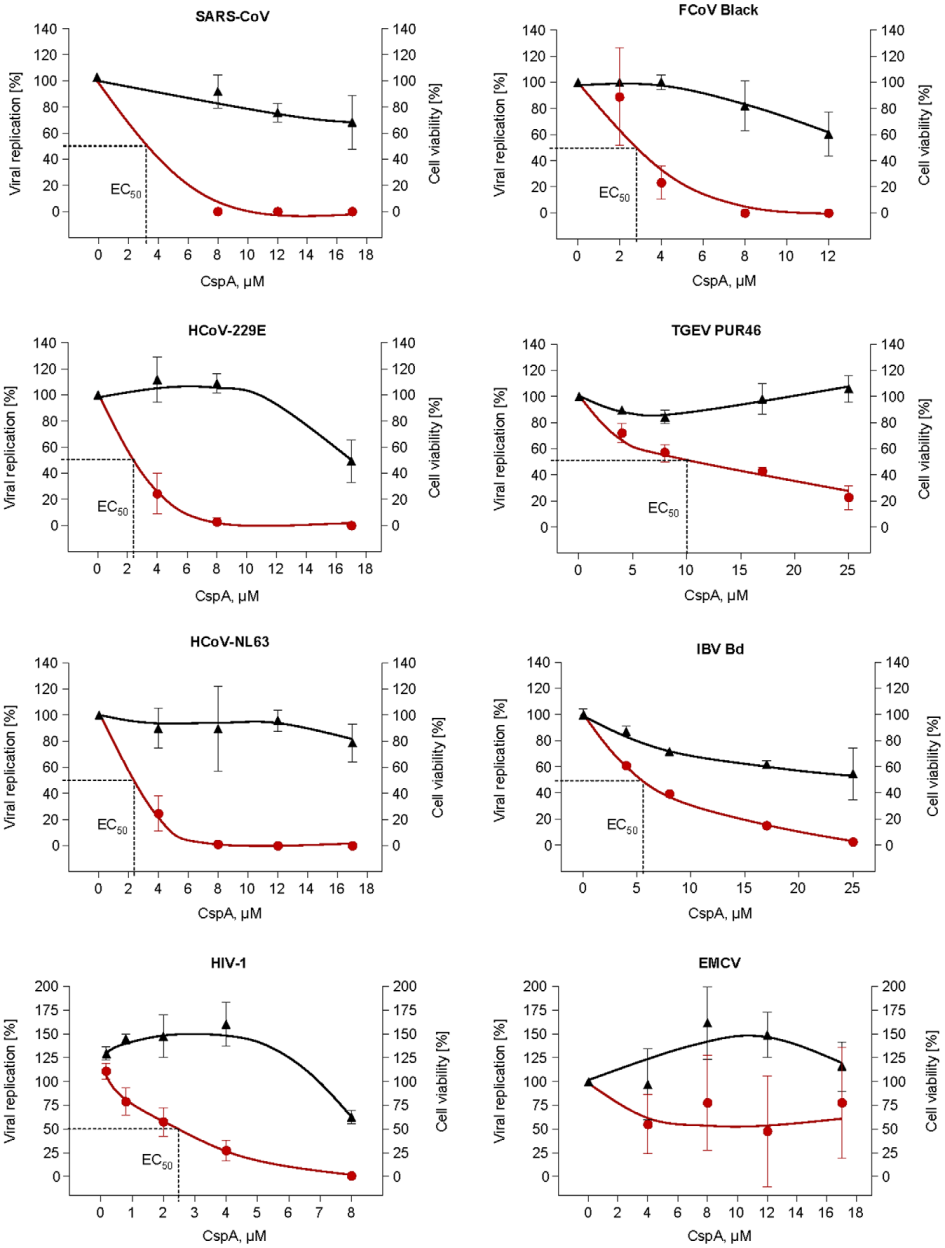
Effect of Cyclosporin A on human (SARS-CoV, HCoV-229E-luc and HCoV-NL63), animal CoV (FCoV, TGEV, IBV) and control virus (HIV-1/EMCV) replication. SARS-CoV, and EMCV were plaque-titrated on VeroE6, IBV-Beaudette in Vero cells, HCoV-NL63 on CaCo-2, TGEV PUR46 on St-cells, FCoV Black and FCoV 791146 on FCWF cells. HCoV-229E-luc was titrated on Huh-7 Lunet and HIV-1 on C8166 SEAP cells. Data shown are mean values of at least three experiments. HIV-1 data show one representative experiment out of three, values are averages of triplicates. Left and right Y-axes represent the percentage of virus replication and cell viability with the mock-treated cells set as 100%, respectively. CspA concentrations used for each virus are given on the x-axis. The graphs were plotted using the Fit Spline algorithm of Prism Software 4.0 (for Mac) of Graphpad Software Inc. The 50% effective dose (EC50) was calculated by regression analysis of the respective virus CPE.

CspA is a highly efficient antagonist of NFAT activation, interacting with cyclophilins. Due to the high specificity of Nsp1-dependent activation of NFAT and due to the high redundancy of SARS-CoV interactions with upstream elements of the CnA/NFAT pathway, we suspected an essential function for the virus. It was therefore investigated whether CspA might influence viral replication (**Figure 8**). Vero cells were inoculated with a low dose of SARS-CoV (MOI = 0.0001) and growth of virus replication was determined by real-time RT-PCR and plaque titration. In parallel cell cultures treated with the same concentrations of CspA, cell viability was measured with a highly sensitive assay based on ATP provision in metabolically active cells. A profound and dose-dependent inhibition of replication of SARS-CoV strain Frankfurt-1 in Vero E6 cells was seen in absence of cytopathic effects conferred by the compound. The 50% effective inhibitory concentration was 3.3 µM.

Because Nsp1 proteins of group I and SARS coronaviruses share structural and functional similarities [22], it was tested whether the inhibitory effect of CspA could be extended to other pathogenic CoVs. These included members of the genera *Alphacoronavirus* (human CoV-NL63 and -229E, Feline CoV serotypes I and II [strains Black and 791146], porcine transmissible gastroenteritis virus [TGEV]), *Betacoronavirus* (SARS-CoV isolates Frankfurt and Hongkong) and *Gammacoronavirus* (avian infectious bronchitis virus [IBV]). All tested CoVs were inhibited by CspA; replication of TGEV and IBV in the tested range (up to 25 µM) was diminished close to background by CspA. HCoV-NL63 and -229E and the two Feline CoV serotypes were completely inhibited, with 50% effective concentrations of 2.3 µM, 2.3 µM and 2.7 µM, respectively (**Figure 8**). **Figure S3** shows reduction of virus replication in a log scale.

### CspA inhibits a SARS-CoV replicon

**Figure 9:**
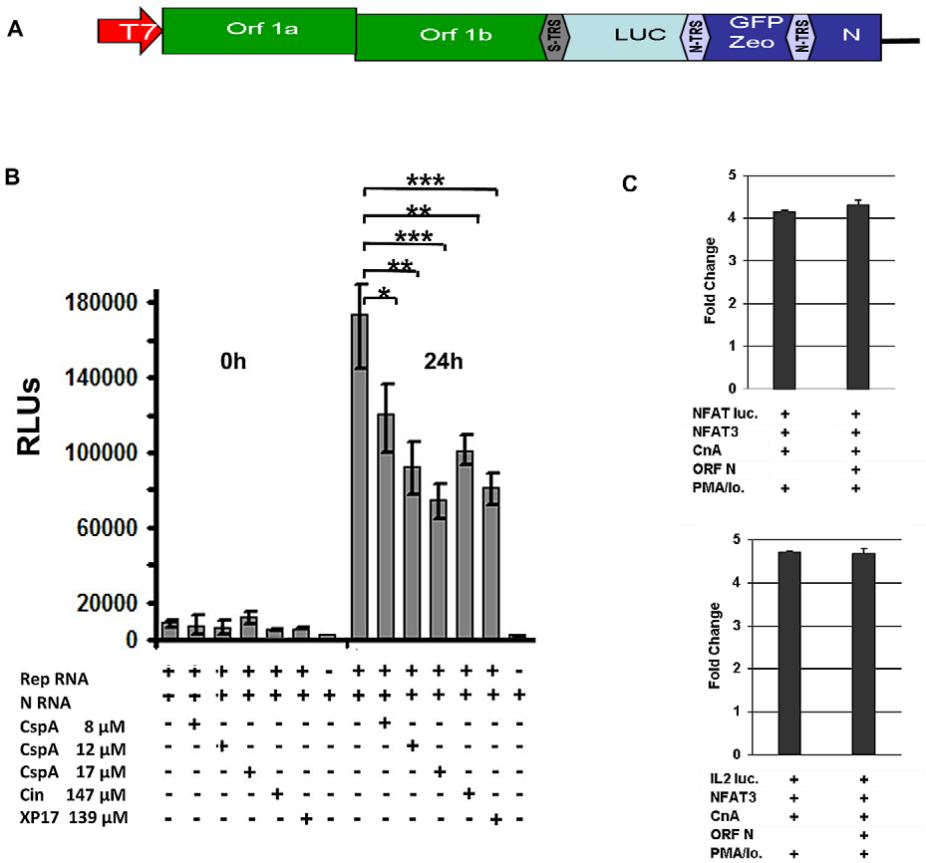
Effect of Cyclosporin A on human SARS-CoV replicon. **A**) Schematic drawing of replicon structure. **B**) Inhibition assay: BHK cells were electroporated in six-well plates with *in vitro* transcribed replicon RNA containing the *Metridia* luciferase gene and N RNA. After 16 hours supernatant was removed and cells were washed twice with PBS. After addition of fresh medium cells were incubated for another 24 hours. Second wash PBS and supernatant taken after 24 hours (50 µl each) were analysed for Luciferase activity. Values are expressed as relative light units (RLU). * *P*<0.05; ** *P*<*0.01,* *** *P*<*0.005.* **C**) HEK293 cells were transiently cotransfected with NFAT or IL-2 reporter plasmid (NFAT luc, IL-2 luc) and expression plasmids encoding NFAT3, Calcineurin (CnA) and SARS-CoV ORF N. l After transfection cells were cultured in absence or presence of the calcineurin stimulators PMA and ionomycin (PMA/Io.)

In order to determine the principal stage of the CoV replication cycle inhibited by CspA, a novel SARS-CoV replicon carrying a secreted *Metridia* luciferase reporter construct instead of the major structural proteins S, E, and M was used (**Figure 9A**). The replicon RNA together with an mRNA for the nucleocapsid protein was electroporated in BHK cells. Replicon activity in parallel reactions was controlled to be at the same level after 16 h of incubation (data not shown), and increasing amounts of CspA were added to cells after repeated washing. As shown in Figure 9B, accumulated luciferase activities in supernatants were decreased in a CspA dose-dependent manner after 24 h. Two different specific inhibitors of the CoV main protease, Cinanserin [23] and XP17 (R. H., own unpublished observations), inhibited replicon activity to a comparable extent as CspA, at comparable substance concentrations (**Figure 9B**). To control against any influence of the nucleocapsid protein that is co-electroporated for maximal replicon efficiency [24], [25], [26] and that is also contained in the replicon RNA, this protein was expressed from a eukaryotic expression vector in the same cells and an NFAT induction assay was conducted as described above. No N-dependent effect on the assay was seen (**Figure 9C**). These results suggest an action of CspA on genome replication and/or transcription, rather than other stages such as virus entry or egress.

## Discussion

Various genomic and proteomic methods have been utilized to identify protein-protein interactions in the context of viral replication [6], [27], [28], [29], [30], [31]. HTY2H is among the most direct approaches to identify interactions between members of viral ORFeomes and large host cDNA libraries. Major advantages of the method include its potential for high throughput testing and automation, as well as its high sensitivity. The latter facilitates investigation of proteins expressed at low levels and of those causing weak and transient interactions [32], [33]. Drawbacks include the inability to control and confirm expression of genes of interest, other than by positive selection of yeast expression clones containing nutritional markers. Moreover, some proteins need posttranslational modifications not provided by the yeast cell, in order to interact with binding partners. Also, since interactions of bait and prey proteins take place in the nucleus of the yeast cell, the assay is influenced by hydrophobic and transmembrane regions affecting the nuclear membrane. It is well known that a considerable fraction of interacting proteins in HTY2H represent false positive findings, making it absolutely necessary to validate interactions by independent eukaryotic assays. We have implemented a version of the Lumier assay that is amenable for screening in mammalian cells at a medium scale of parallelity [34]. Our modified version using a protein A tag instead of a Flag tag enables automated capture of precipitates on IgG Fc-coated magnetic beads. Throughput is mainly limited by the requirement to subclone Y2H plasmid inserts, as the assay does not involve any cell-based imaging or other readouts going beyond *in-vitro* Luciferase assays. In our analysis of 86 Y2H-positive interaction partners we achieved a positive confirmation rate of about 42% in category A and B interactors, which is in good agreement with a recent standardized comparison of five different interaction assays in which the LUMIER pull-down assay showed the highest sensitivity (36%) on a positive reference set of human proteins [10]. It has to be mentioned that all interaction assay systems carry intrinsic limitations. In the case of our modified Lumier method the Renilla and protein A tags are rather long as compared to His or HA tags. Therefore, true interactions might be missed, and it is possible that the interaction of PPIA with full-length nsp1 is sterically prevented by the length of these tags, as compared to the N-terminal fragment of Nsp1. But the chief attraction of our method is its applicability in high thoughput assays.

The range of interactors identified in this study defines an unprecedented resource for future investigations into pathogenetic mechanisms and antiviral applications against CoVs. In order to demonstrate that HTY2H can afford a direct identification of novel antiviral targets, we have chosen one promising group of interactors for further investigation in the present study. The interaction of Nsp1 with the cyclophilins PPIA, PPIB, PPIG, PPIH, the FK506-binding proteins FKBP1A/B, and the CnA (calcipressin) regulators RCAN1 and RCAN3 represented a highly redundant virus-host interaction involving critical elements of the same regulatory network immediately upstream of the CnA/NFAT pathway. Nsp1 is a virulence factor *in-vivo* whose action has been linked with early stages of the immune response, including antagonism against IFN signaling and inhibition of host protein synthesis [35], [36], [37], [38]. Our findings add an important new dimension to Nsp1′s role in pathogenicity, identifying this protein as a strong and specific activator of NFAT enhancing the induction of the IL-2 promoter. The increase of NFAT activation extended to all three major NFAT species, suggesting a potential for induction of broad and systemic cytokine dysregulation by affecting several types of immune cells. The pattern of cytokine dysregulation in severe SARS cases differed from the cytokine burst seen in other acute viral diseases in its delayed occurrence, manifesting beyond the second week of symptoms. Interestingly, it was noted upon clinical observations that late aggravation was correlated with severe clinical outcome, triggering parallel efforts in several centers to treat patients empirically with steroids [39], [40], [41]. Our results suggest an influence on fundamental triggers of immune cell activation, contributing an explanation for the cytokine dysregulation and immune-dependent pathogenesis observed in severe cases of SARS.

The detected interactions with immunophilins caught our attention because peptide inhibitors such as CspA and Tacrolimus (FK506) are available that bind to immunophilins and cause dramatic effects on the cellular phosphatase CnA, suppressing the CnA/NFAT immune-regulatory pathway [42]. Cyclophilins are essential cofactors for replication of HCV, HIV and of some parasites [43]. Incorporation of PPIA into HIV-1 particles via binding to *gag* is paralleled by its binding to the N protein of SARS-CoV by an educated guess finding using surface plasmon resonance biosensor technology [44]. Furthermore, in a recent proteomics study in which viral and cellular proteins incorporated into SARS-CoV virions were spectrometrically profiled PPIA was also found in purified virus particles [31]. We have indeed shown that the inhibition of cyclophilins by CspA exhibited strong and specific inhibitory effects on members of all three genera of CoVs. This broad antiviral action is supported by a recent characterization of Nsp1 structural conservation that extends beyond the limits of CoV genera [22]. Inhibition took place in the low micromolar range, indicating prospects for future investigation of similar (non-immunosuppressive) drugs as broad-range antivirals. Such drugs have already been employed against HCV, whose nonstructural NS5A and NS2 proteins interact with cyclophilins and whose replication can be inhibited by CspA and non-immunosuppressive derivatives thereof [45].

Our study is limited in that it does not clarify the biological functions of the interaction between Nsp1 and its cellular partners, nor does it confirm the involvement of Nsp1 in full virus context by knock out experiments in recombinant viruses. Brockway and Denison showed that deletion of residues in the amino-terminal half of nsp1 is not tolerated for a productive infection [46]. Thus it seems not possible to construct viruses with mutations in the N- terminal half of nsp1 which do not bind to cyclophilins anymore. It will thereofore be difficult to exactly delineate the role of nsp1 in virus-host cooperation. It should also be appreciated that CspA-inhibitable NFAT induction by SARS-CoV nsp1 may be independent of the replication inhibition by CspA. CspA specifically binds to cyclophilins and this complex binds to calcineurin phosphatase preventing the dephosphorylation of NFAT. Binding of nsp1 to cyclophilins and the induction of NFAT is obviously inhibited by Csp A. On an independent level, formation of the Cyclophilin-CspA complex might prevent those cyclophilin functions required for virus replication. Complex further studies involving multiple CoV systems will be required to delineate these functions.

Nevertheless, we have been able to shed more light on the principal stage of the virus replication cycle that is subject to CspA-dependent inhibition. Our experiments using a SARS-CoV replicon suggest strongly that the processes afforded by the replicative proteins rather than stages of virus entry and egress, are affected. Further experiments need to be done in the future in order to investigate on a mechanistic level the potential breadth of the identified antiviral effect. In particular, the large diversity of CoVs in animal reservoirs generates interest in studying how conserved this particular virus-host interaction might be between non-human CoVs and human cells, and whether this could be exploited as a truly broad-range antiviral target that covers epidemic and reservoir-borne viruses alike.

## Materials and Methods

### Cells

HEK293, HEK293lp (low passage), Vero E6, CaCo-2, HRT 18, Huh 7, FCWF- and St-cells were grown in Dulbecco’s modified Eagle medium (DMEM) containing 10% FBS, 1% L-glutamine, 1% penicillin/streptomycin and 1% non essential amino acids. C8166 SEAP cells were cultured in RPMI medium with 10% FBS and 1% penicillin/streptomycin. Jurkat cells were propagated in RPMI-medium containing 10% FBS, 1% L-glutamine and 1% penicillin/streptomycin.

### Y2H library screening

Cloning of the SARS ORFeome into destination bait vector pGBKT7-DEST for Y2H screening was described previously [6]. A series of SARS-CoV (Frankfurt isolate) bait clones containing ORF fragments depleted of transmembrane regions were generated in addition (primers and cloning procedures available on request). Automated yeast two-hybrid screens were essentially done as described previously [7], [47], with the following modifications. Human cDNA libraries from human brain and fetal brain (Clontech) as well as a library of individually cloned full–length open reading frames from cDNAs of 5000 different genes were screened to a minimal coverage of 5 million clones per library. To mate yeast strains, exponentially growing cultures at an OD_600nm_ of 1 were combined, pelleted by centrifugation, and resuspended in an equal volume of YPDA (Yeast Extract Peptone Dextrose Adenine) containing 20% PEG 6000. For the generation of a high-confidence dataset, interaction pairs were selected which were isolated at least twice, or where the bait interacted with two highly related preys, and which did not involve promiscuous preys.

### Modified LUMIER assays

For LUMIER assays, proteins were transiently expressed in HEK293 cells as N-terminal fusion proteins with the *Staphylococcus aureus* protein A tag or *Renilla reniformis* luciferase. 20 ng of each expression construct were transfected into 10,000 HEK293 cells using 0.05 µl of lipofectamine 2,000 (Invitrogen) in 96 well plates. After 40 hours, medium was removed and cells were lysed on ice in 10 µl of ice-cold lysis buffer (20 mM Tris pH 7.5, 250 mM NaCl, 1% TritonX-100, 10 mM EDTA, 10 mM DTT, Protease Inhibitor Cocktail [Roche # 1 836 170], Phosphatase Inhibitor Cocktail [Roche, # 4 906 837], Benzonase [Novagen #70746, 0,0125 units per µl final concentration]) containing sheep-anti-rabbit IgG-coated magnetic beads (Invitrogen, Dynabeads M280, 2 mg/ml final concentration). Lysates were incubated on ice for 15 minutes. 100 µl of washing buffer (PBS, 1 mM DTT) were added per well, and 10% of the diluted lysate was removed to determine the luciferase activity present in each sample before washing. The rest of the sample was washed 6 times in washing buffer in a Tecan Hydroflex plate washer. Luciferase activity was measured in the lysate as well as in washed beads. Negative controls were transfected with the plasmid expressing the luciferase fusion protein and a vector expressing a dimer of protein A.

For each sample, four values were measured: the luciferase present in 10% of the sample before washing, the luciferase activity present on the beads after washing, and the same values for the negative controls. Normalised interaction signals were calculated as follows: Log(bound)/log(input) – log(bound nc)/log(input nc). Z-scores were calculated by subtracting the mean and dividing by the standard deviation. The mean and standard deviation were calculated from large datasets of protein pairs which were not expected to interact, i.e. from negative reference sets. Normalised signal to noise ratios were calculated as follows: (bound/input)/(bound nc/input nc)[10].

### Reporter gene assays

For reporter gene expression HEK293 cells were transiently transfected with 2 µg DNA containing 460 ng of the respective expression plasmids encoding NFAT, calcineurin, SARS-CoV Nsp1 and reporter genes as indicated (six well plates). DNA was transfected using FuGENE HD reagent (Roche Applied Science). For reporter gene expression in Jurkat cells (1×10^6^ cells) 1 µg reporter plasmid and 1 µg expression plasmid encoding SARS-CoV Nsp1 were transiently cotransfected using the Amaxa Cell Line Nucleofector Kit (Lonza). For reporter gene assays in the viral context HEK293lp cells (24-well plates) were transiently transfected with 500 ng reporter plasmid using Lipofectamine LTX (Invitrogen) according to the manufacturer’s instructions. 24 h post transfection cells were infected with SARS-CoV HK at an MOI = 1. 19 h after transfection cells were harvested and Promega’s dual luciferase assays were performed according to the manufacturer’s instructions. All results were normalized to a simultaneously transfected *Renilla* luciferase (pRL-null, Promega). Cells were stimulated by PMA (40 ng/ml) and Ionomycin (2 µM) in culture medium. The NFAT-pathway was inhibited by Cyclosporin A (50 ng/ml). Virus infected cells were lysed 17 h post infection with Promega passive lysis buffer (PLB) and 1% Igepal (Sigma-Aldrich). All experiments were repeated at least three times.

### Virus inhibition experiments and plaque titration

#### SARS - CoV

Vero E6 cells were seeded in 6-well plates. At a confluence of 80%, cells were washed with phosphate buffered saline (PBS) and infected with a MOI = 0.0001. After 1 h adsorption cells were washed twice with PBS and supplemented with 2 ml fresh media and inhibitor in different concentrations. Supernatants were tested after 24 h p.i. and 48 h p.i.. For quantitative real time RT-PCR 140 µl supernatant were taken and analysed as described previously [48]. Plaque titrations on Vero E6 cells were performed using Avicel overlays (RC581, FMC BioPolymer, Belgium) as described [18].

#### HCoV-NL63

CaCo-2 cells were seeded in 24-well plates. For quantitative real time RT-PCR cells were infected with a MOI = 0.004 at a confluence of 100%. After 1 h adsorption the viral inoculum was removed, cells were washed twice with PBS and 1 ml fresh medium supplemented with different inhibitor concentrations was added to the cells. 70 µl supernatant were tested on day 2 and day 4 p.i. Viral RNA was extracted from cell culture supernatant with the QIAamp Viral RNA mini Kit (QIAGEN, Hilden, Germany). Real time RT-PCR and determination of viral replication by plaque assay was done as described previously [49]. Various inhibitor concentrations were directly added to the overlay medium. Cells were fixed and plaques were stained with 0.2% crystal violet, 11% formaldehyde, 2% ethanol, and 2% paraformaldehyde.

#### HCoV-229E

Huh-7 cells were seeded in 24-well plates and incubated until the monolayer was 70–80% confluent. Cells were infected with HCoV229E-luc (V. Thiel unpublished data), in which ORF 4 is replaced by *Renilla* luciferase, at an MOI = 0.1. After 1 h adsorption viral inoculum was removed, cells were washed with PBS and incubated with 1 ml fresh medium containing different inhibitor concentrations. Viral replication was determined 24 h and 48 h p.i. by *Renilla* Luciferase Assay System (Promega, Madison, USA) according to the manufacturer’s instructions.

#### IBV Beaudette

Vero cells grown on 24-well plates were infected with IBV Beaudette at an MOI = 0.00025. Methylcellulose was added after 1 h incubation at 37°C. Different concentrations of CspA at a range from 0 to 25 µM were mixed with the methylcellulose. After 24 h, the cells were fixed with 3% paraformaldehyde. Plaques were stained with an anti-IBV polyclonal serum raised in rabbits and a FITC-labelled secondary antibody.

#### TGEV PUR46

St cells were seeded in 6-well plates. At a confluence of 100%, cells were washed twice with PBS and infected with a MOI = 0.000066. Plaque assays were performed as described previously [50]. Different inhibitor concentrations were directly added to overlay media.

#### FCoV Black (Serotype I) and 791146 (Serotype II)

One day before the experiment 1.5 * 10^6^ FCWF cells were seeded in 12-well plates. Cells were infected with respective virus dilutions. 1 h after infection cells were washed with 1 ml PBS and 2 ml fresh medium with different inhibitor concentrations were added. 48 h p.i. supernatants were analyzed via plaque assay on freshly seeded FCWF cells with 100% confluence. Again, cells were infected for 1 h, after washing with 1 ml PBS, 1 ml 1% Carboxymethylcellulose medium was added to cells. After 72 h the plaques were analyzed.

#### EMCV

Vero E6 cells were seeded in 6-well plates and infected with an MOI = 0.01. After 1 h infection medium was removed and replaced by fresh medium supplemented with different inhibitor concentrations. 24 h p.i. supernatants were analyzed by plaque titration on freshly seeded Vero E6 cells. Cells were infected for 1 h, washed with 1 ml PBS and incubated with 0.4% Noble Agar (Difco) overlay medium for 72 h. After fixation with 2 ml 5% tri-chloro acetic acid (TCA) for 5 min, plaques were stained with 1% crystal violet in 3.6% formaldehyde, 1% methanol, and 20% ethanol.

#### HIV-1

C8166 SEAP cells were seeded at a density of 30,000 cells per 96-well and CspA was added to a final concentration of 0.2, 0.4, 0.8, 2, 4, and 8 µM. Cultures were inoculated with HIV-1 NL4-3 at low MOI that allowed complete inhibition of replication in the presence of the HIV protease inhibitor Nelfinavir (NFV) to ensure multiple rounds of infection. Half of the culture medium was discarded on day 3 and replaced by fresh medium ± inhibitor. HIV-1 replication was monitored microscopically via cytopathic effects and measured on day 5 via the activity of secreted alkaline phosphatase (SEAP) in the cell culture supernatant employing the Phospha-Light SEAP Reporter Gene Assay (Applied Biosystems).

In all experiments the outcome of the mock-treated cells were set as 100%. Data shown are the mean values of at least three experiments. HIV-1 data show one representative experiment out of three, values are averages of triplicates. DMSO was always added as a control corresponding to the highest inhibitor concentration.

### Cytotoxicity assay

Cytotoxicity tests of all cell lines were carried out in a 96-well format with the *CellTiter-Glo Luminescent Cell Viability Assay* (Promega, Madison,USA) according to the manufacturer’s instructions.

### SARS replicon and inhibitor assay

#### SARS-CoV replicon

The SARS replicon was based on our previously pubished infectious SARS-CoV cDNA clone [51]. By PCR mutagenesis and classical cloning techniques ORFs 2–8 were deleted or replaced by marker genes, respectively. In ORF 2 (spike gene) a secreted *Metridia* Luciferase (Promega) was inserted, using the original transcription-regulating sequence. Furthermore, for potential selection, a GFP-Zeocin fusion protein was placed in open reading frame 3a. Cloning details will be given by authors upon request.

#### Inhibitor assays

All assays were performed in triplicates. Replicon RNA and N RNA were transcribed and electroporated in BHK cells as described [51]. For inhibitor assays, cells were seeded in 6 well plates after electroporation using 2×10^4^ cells per well and incubated for 16 hours at 37°C. Hereafter, supernatant was removed entirely and cells were washed twice with PBS. Then 2 ml of fresh medium with indicated concentration of inhibitors were added. For each timepoint 50 µl of supernatant were removed for Luciferase assay (Promega). RLUs were measured as recommended by the manufacturer.

### Gene Ontology (GO) over-representation analysis

To determine which cellular pathways are targeted by SARS-CoV, functional categories enriched among SARS cellular interaction partners were identified using a Gene Ontology (GO) over-representation analysis. For this purpose, p-values were determined with the hypergeometric test implemented in the Ontologizer software [12]. P-Values were corrected for multiple testing using the FDR-method by Benjamini and Hochberg [52] and significant terms were identified at a threshold of 0.05.

### Analysis of protein complexes

We analyzed whether proteins involved in protein complexes were preferentially targeted by SARS-CoV proteins and which complexes were preferentially targeted. For this purpose, protein complexes for humans were extracted from the CORUM database[53]. After removing complexes which were identical to another complex, we obtained a data set of 1184 complexes containing 2079 distinct proteins. P-values for the enrichment of protein complex subunits among SARS targets were calculated with the hypergeometric test assuming a background of ∼ 25,000 proteins (Table S4). We furthermore analyzed 9 complexes which targeted at least four subunits by SARS proteins. Using a hypergeometric test, we determined p-values for the enrichment of SARS target proteins among the subunits of each complex. P-values were corrected for multiple testing using the FDR- method by Benjamini and Hochberg (Table S5).

### Centrality of virus targets

Interactions between SARS proteins and human proteins were connected to a network of human protein-protein interactions taken from the Human Protein Reference Database (HPRD, Release 7) [54] and the Biological General Repository for Interaction Datasets (BioGRID) database [55]. We then compared the distribution of degree (number of interactions) and betweenness centrality [13] for the viral targets against all other proteins in the human networks with the Kolmogorov-Smirnov test in R [56]. P-Values were again corrected for multiple testing using the FDR-method by Benjamini and Hochberg. Degree and betweenness centrality are alternative measures of network centrality for individual proteins. High degree characterizes so-called hubs which are highly interactive while high betweenness centrality characterizes so-called bottlenecks which are central to many connections between proteins.

## Supporting Information

Figure S1 **Level 2 virus-host high-confidence network.** Interactions are shown between the viral proteins, the direct cellular interaction partners of SARS proteins (level 1) and the interaction partners of the direct cellular interaction partners (level 2). Colors are as follows: Blue for viral proteins, orange for direct cellular interaction partners and yellow for cellular proteins interacting with a cellular target of SARS. Viral-host interactions are shown in black, and intraviral and intra-host interactions in grey.
(TIF)

Figure S2 **Targeting of hubs and bottlenecks.** Interactions between SARS proteins and human proteins were connected to a network of human protein-protein interactions taken from the Human Protein Reference Database (HPRD) (Peri et al. 2004) (Release 7) and the Biological General Repository for Interaction Datasets (BioGRID) database (Breitkreutz et al. 2008) (downloaded March, 17, 2009, version 2.0.50). High degree (**A**) and high betweenness centrality (**B**) characterize so-called highly interactive hubs and so-called bottlenecks which are central to many connections between proteins, respectively. FDR-corrected p-values for the difference between target and non-target proteins are provided on top of the bars for the target proteins.
(TIF)

Figure S3 **Log reduction of virus replication.** Values are given in log scale at the indicated cyclosporine A concentrations. Starting titers of the different viruses were all different, i.e. in the case of a low starting titer the drop is not as prominent as in infections with high starting titers. Thus, the drop of titers in log scale can not be compared directly.
(TIF)

Table S1 **Category 1 (A) and category 2 (B) interaction partners of SARS-CoV nsp1 and cellular proteins identified by HTY2H and validated by LUMIER assay.** Of 44 of the high-confidence (A) Y2H interactions that were re-tested in LUMIER assays, 21 (48%) were clearly positive. In contrast, when 42 of the low-confidence Y2H-interactions (category B) were tested in LUMIER assays, a much lower percentage of pairs gave interactions signals above background. For comparison, a negative reference set of 85 random proteins yielded interaction signals which roughly corresponded to the statstically expected numbers for normally distributed signals. A comparison of Braun et al. (see main text) have recently shown that roughly one third of interactions selected from the scientific literature score positive in the LUMIER assays. We therefore estimate the false positive rate of the interactions from our dataset to be in the range of 20-30%. A graphical comparison of these data to a negative control set is depicted in Figure 1.
(DOC)

Table S2 **Identification of previously published SARS-CoV interactions with cellular proteins.** Literature interactions were identified using a combination of text mining and manual curation. Abstracts on SARS containing a human protein and a mentioning of experimental methods such as yeast two-hybrid, Co-Immunoprecipitation or GST pulldown assay were manually screened for interactions between a human and a SARS protein. In the same way, human proteins enriched in SARS abstracts were investigated for interactions. In this way, 28 known interactions between SARS proteins and their human interaction partners were identified. “Y2H this study” (last column) hits refer to human genes identified here and in the literature.
(DOC)

Table S3 **Screening of more than 5,000 abstracts with a human synonym protein list (31,941 entries) on SARS coronavirus using the Text-Mining program** ***syngrep*** **for the occurrence of human targets of SARS proteins.** Interaction partners of SARS-CoV identified in this study are enriched for proteins associated with SARS infection in previous studies. Numbers mean that e.g. in the case of 100 human protein synonyms six SARS or coronavirus protein entries were found.
(DOC)

Table S4 **Gene Ontology over-representation analysis performed on high confidence nsp1-targets.** Among them were five proteins displaying peptidyl-prolyl cis-trans isomerase activity. BH  =  Multiple testing correction with Benjamine-Hochberg. P value cutoff was 0.05.
(DOC)

Table S5 **Protein complexes preferentially targeted by SARS proteins.** Shown are the four significantly enriched SARS-CoV targeted protein complexes.
(DOC)

Table S6 **Protein complexes preferentially targeted by SARS proteins.** Frequency of SARS targets within protein complexes was compared to the overall frequency of protein subunits and was found to be significantly enriched compared to the overall background.
(DOC)
