# Supplementary figures and images for: The SARS-Coronavirus-Host Interactome: Identification of Cyclophilins as Target for Pan-Coronavirus Inhibitors
Source: PLoS Pathog. 2011 Oct 27;7(10):e1002331. doi: 10.1371/journal.ppat.1002331 (PMC3203193; doi:10.1371/journal.ppat.1002331)

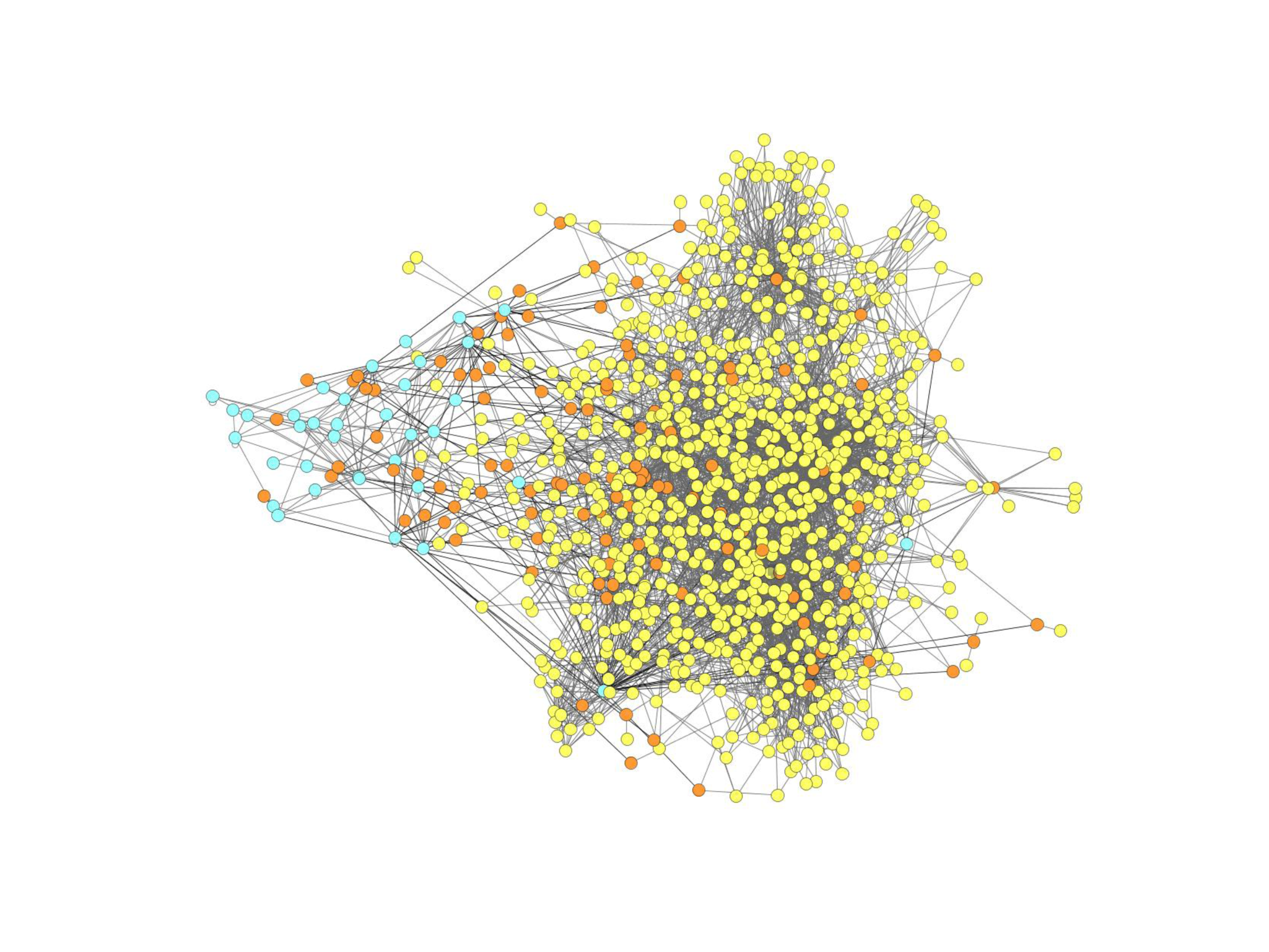

Supplement: Figure S1 — Level 2 virus-host high-confidence network. Interactions are shown between the viral proteins, the direct cellular interaction partners of SARS proteins (level 1) and the interaction partners of the direct cellular interaction partners (level 2). Colors are as follows: Blue for viral proteins, orange for direct cellular interaction partners and yellow for cellular proteins interacting with a cellular target of SARS. Viral-host interactions are shown in black, and intraviral and intra-host interactions in grey. (TIF) [file ppat.1002331.s001.tif]

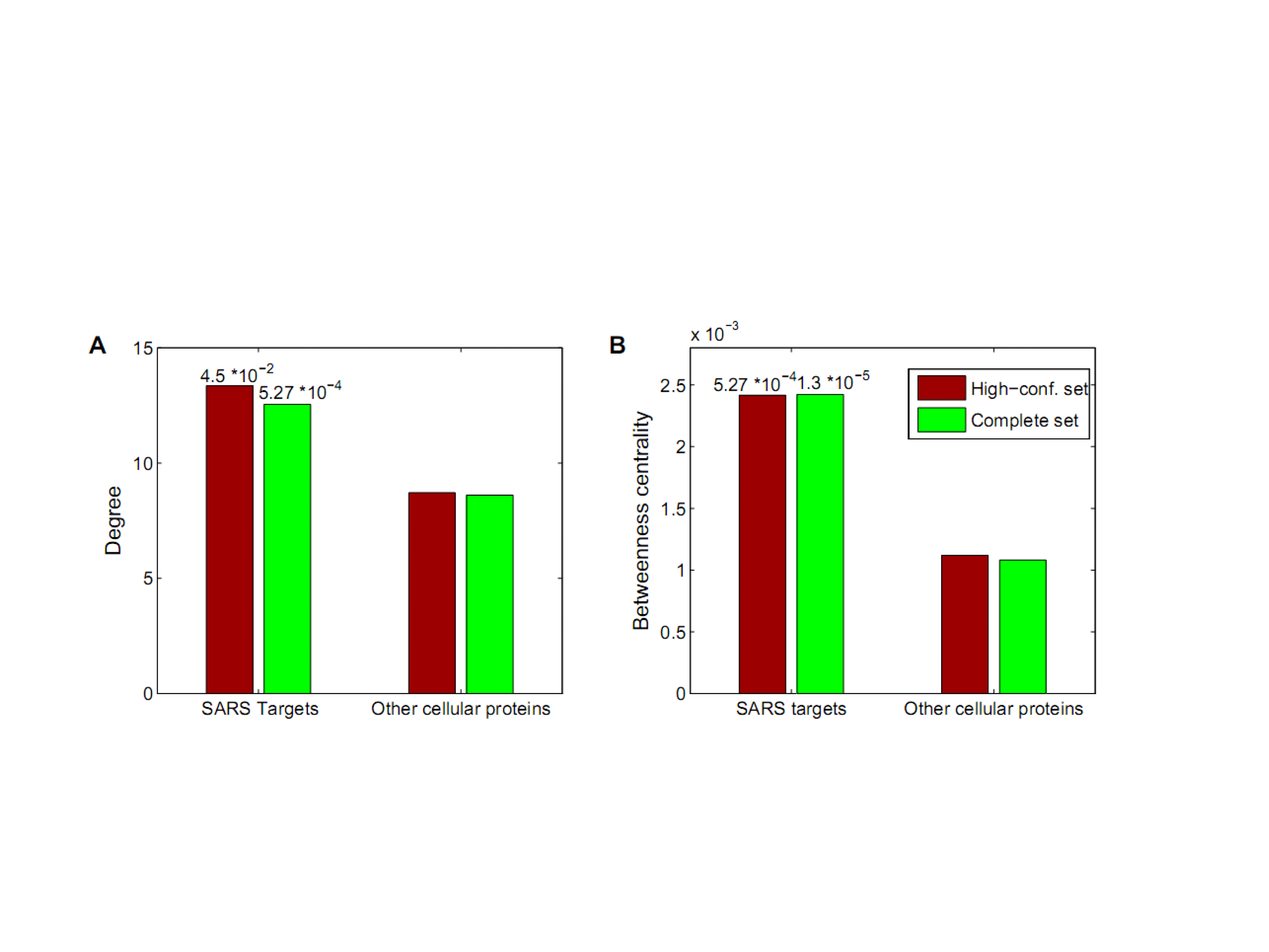

Supplement: Figure S2 — Targeting of hubs and bottlenecks. Interactions between SARS proteins and human proteins were connected to a network of human protein-protein interactions taken from the Human Protein Reference Database (HPRD) (Peri et al. 2004) (Release 7) and the Biological General Repository for Interaction Datasets (BioGRID) database (Breitkreutz et al. 2008) (downloaded March, 17, 2009, version 2.0.50). High degree (A) and high betweenness centrality (B) characterize so-called highly interactive hubs and so-called bottlenecks which are central to many connections between proteins, respectively. FDR-corrected p-values for the difference between target and non-target proteins are provided on top of the bars for the target proteins. (TIF) [file ppat.1002331.s002.tif]

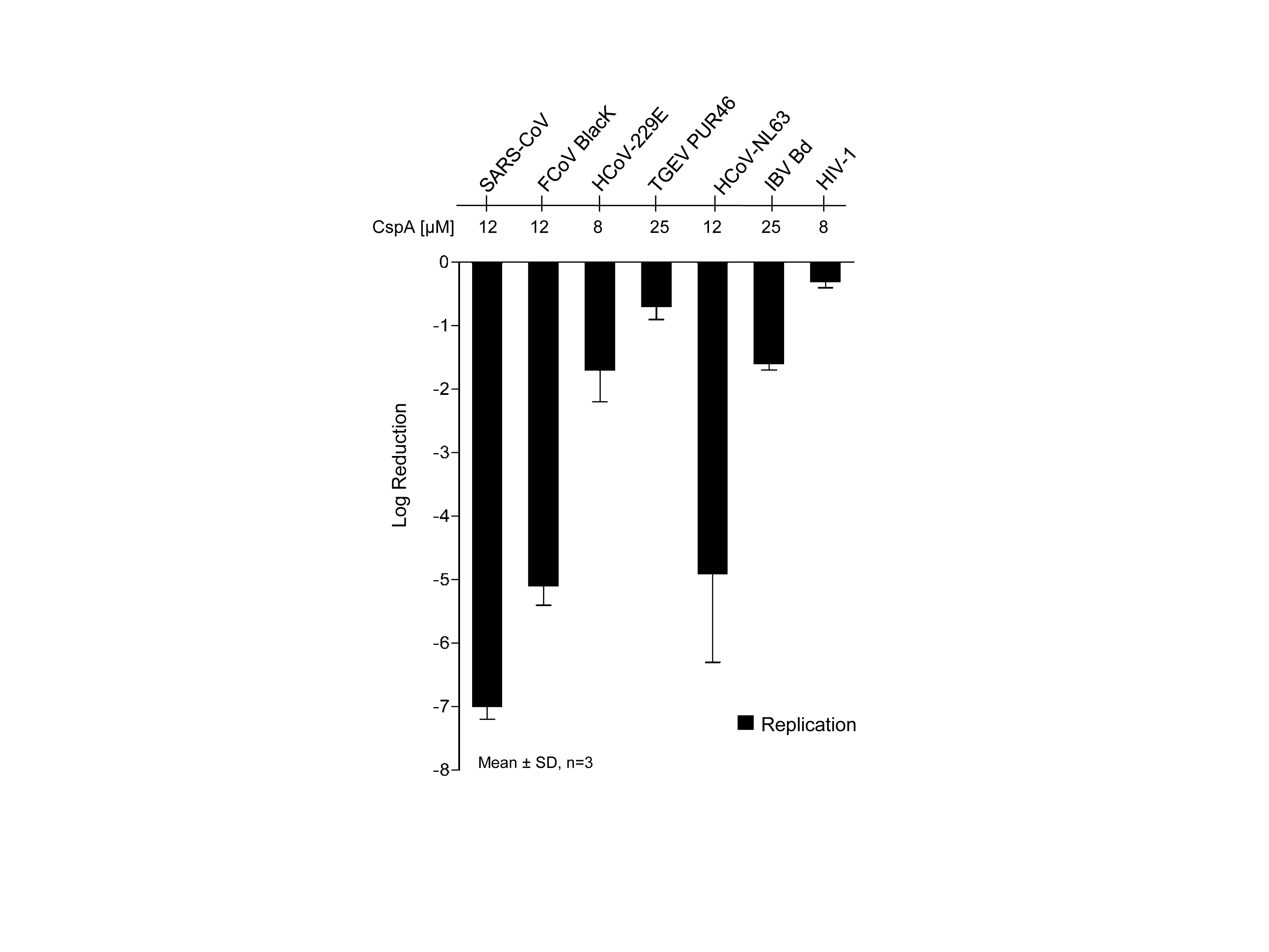

Supplement: Figure S3 — Log reduction of virus replication. Values are given in log scale at the indicated cyclosporine A concentrations. Starting titers of the different viruses were all different, i.e. in the case of a low starting titer the drop is not as prominent as in infections with high starting titers. Thus, the drop of titers in log scale can not be compared directly. (TIF) [file ppat.1002331.s003.tif]
