# Supplementary material for: The SARS-Coronavirus-Host Interactome: Identification of Cyclophilins as Target for Pan-Coronavirus Inhibitors
Source: PLoS Pathog. 2011 Oct 27;7(10):e1002331. doi: 10.1371/journal.ppat.1002331 (PMC3203193; doi:10.1371/journal.ppat.1002331)
Supplement: Table S1 — Category 1 (A) and category 2 (B) interaction partners of SARS-CoV nsp1 and cellular proteins identified by HTY2H and validated by LUMIER assay. Of 44 of the high-confidence (A) Y2H interactions that were re-tested in LUMIER assays, 21 (48%) were clearly positive. In contrast, when 42 of the low-confidence Y2H-interactions (category B) were tested in LUMIER assays, a much lower percentage of pairs gave interactions signals above background. For comparison, a negative reference set of 85 random proteins yielded interaction signals which roughly corresponded to the statstically expected numbers for normally distributed signals. A comparison of Braun et al. (see main text) have recently shown that roughly one third of interactions selected from the scientific literature score positive in the LUMIER assays. We therefore estimate the false positive rate of the interactions from our dataset to be in the range of 20-30%. A graphical comparison of these data to a negative control set is depicted in Figure 1. (DOC) [file ppat.1002331.s004.doc]

| **Category** | **Bait** | **Prey gene symbol** | **Prey gene id** | **Number of times this  prey has been isolated.** | **Description** | **Library for Y2H screening** | **LUMIER z-scores** | **signal to background ratios** |
| --- | --- | --- | --- | --- | --- | --- | --- | --- |
| A | ORF13 | RYBP | 23429 | 5 | RING1 and YY1 binding protein | ORFeome | 4,9 | 78,4 |
| A | NSP1 | PPIA | 5478 | 1 | peptidylprolyl isomerase A (cyclophilin A) | fetal brain | 3,2 | 20,2 |
| A | NSP8 | NOMO3 | 408050 | 2 | NODAL modulator 3 | brain | 2,9 | 10,4 |
| A | NSP1 | FKBP1A | 2280 | 1 | FK506 binding protein 1A, 12kDa | fetal brain | 2,9 | 19,0 |
| A | NSP1 | PPIG | 9360 | 1 | peptidylprolyl isomerase G (cyclophilin G) | brain | 2,8 | 13,0 |
| A | ORF9b | MARK3 | 4140 | 1 | MAP/microtubule affinity-regulating kinase 3 | fetal brain | 2,7 | 13,0 |
| A | NSP1 | PPIH | 10465 | 1 | peptidylprolyl isomerase H (cyclophilin H) | fetal brain | 2,7 | 11,5 |
| A | NSP1 | RCAN3 | 11123 | 1 | RCAN family member 3 | ORFeome | 2,5 | 8,3 |
| A | ORF14 | HGS | 9146 | 6 | hepatocyte growth factor-regulated tyrosine kinase substrate | ORFeome | 2,4 | 9,2 |
| A | ORF8a | BAT3 | 7917 | 3 | HLA-B associated transcript 3 | fetal brain | 2,0 | 6,7 |
| A | NSP7 | DDAH2 | 23564 | 13 | dimethylarginine dimethylaminohydrolase 2 | fetal brain | 1,8 | 7,5 |
| A | ORF7b | CAMLG | 819 | 2 | calcium modulating ligand | fetal brain | 1,5 | 6,3 |
| A | NSP12 | CHMP2B | 25978 | 15 | chromatin modifying protein 2B | ORFeome, brain | 1,3 | 4,2 |
| A | NSP13 | RYBP | 23429 | 5 | RING1 and YY1 binding protein | ORFeome | 1,3 | 5,3 |
| A | NSP12 | C1orf142 | 116841 | 3 | chromosome 1 open reading frame 142 | fetal brain | 1,3 | 3,1 |
| A | Nsp3 | MKRN2 | 23609 | 131 | makorin, ring finger protein, 2 | ORFeome, brain, fetal brain | 1,1 | 3,3 |
| A | NSP12 | TPSAB1 | 7177 | 2 | tryptase alpha/beta 1 | ORFeome | 1,1 | 5,0 |
| A | NSP14 | SERPING1 | 710 | 15 | serpin peptidase inhibitor, clade G (C1 inhibitor), member 1, (angioedema, hereditary) | brain | 1,1 | 3,2 |
| A | Nsp3 | MKRN3 | 7681 | 8 | makorin, ring finger protein, 3 | ORFeome | 1,1 | 3,4 |
| A | NSP9 | CHMP2B | 25978 | 2 | chromatin modifying protein 2B | fetal brain | 1,1 | 4,8 |
| A | ORF9b | PSMA2 | 5683 | 4 | proteasome (prosome, macropain) subunit, alpha type, 2 | fetal brain | 1,1 | 3,5 |
| A | NSP9 | C1orf63 | 57035 | 2 | chromosome 1 open reading frame 63 | brain, fetal brain | 0,9 | 2,8 |
| A | NSP3 | ZNF410 | 57862 | 1 | zinc finger protein 410 | fetal brain | 0,9 | 2,2 |
| A | NSP12 | POLR2B | 5431 | 2 | polymerase (RNA) II (DNA directed) polypeptide B, 140kDa | brain | 0,9 | 4,0 |
| A | NSP2 | DEDD2 | 162989 | 3 | death effector domain containing 2 | brain | 0,9 | 2,7 |
| A | NSP14 | DCTN2 | 10540 | 4 | dynactin 2 (p50) | fetal brain | 0,7 | 2,4 |
| A | NSP9 | NPHP3 | 27031 | 2 | nephronophthisis 3 (adolescent) | fetal brain | 0,7 | 2,6 |
| A | NSP12 | TPSB2 | 64499 | 1 | tryptase beta 2 | ORFeome | 0,7 | 1,6 |
| A | ORF3b | DCTN2 | 10540 | 2 | dynactin 2 (p50) | fetal brain | 0,6 | 2,1 |
| A | NSP7 | RNF128 | 79589 | 3 | ring finger protein 128 | fetal brain | 0,6 | 2,4 |
| A | NSP7 | BAP1 | 8314 | 2 | BRCA1 associated protein-1 (ubiquitin carboxy-terminal hydrolase) | fetal brain | 0,6 | 1,8 |
| A | ORF14 | SERPING1 | 710 | 36 | serpin peptidase inhibitor, clade G (C1 inhibitor), member 1, (angioedema, hereditary) | fetal brain | 0,4 | 1,9 |
| A | NSP7 | ARL4D | 379 | 2 | ADP-ribosylation factor-like 4D | brain, fetal brain | 0,4 | 2,1 |
| A | NSP12 | CCHCR | 54535 | 7 | coiled-coil alpha-helical rod protein 1 | ORFeome | 0,3 | 1,8 |
| A | NSP9 | FAHD1 | 81889 | 4 | fumarylacetoacetate hydrolase domain containing 1 | brain, fetal brain | 0,2 | 1,1 |
| A | NSP8 | BZW2 | 28969 | 2 | basic leucine zipper and W2 domains 2 | brain | 0,1 | 2,3 |
| A | NSP7 | NCOA5 | 57727 | 2 | nuclear receptor coactivator 5 | fetal brain | 0,0 | 1,5 |
| A | ORF3b | SERPING1 | 710 | 39 | serpin peptidase inhibitor, clade G (C1 inhibitor), member 1, (angioedema, hereditary) | fetal brain, ORFeome | 0,0 | 1,2 |
| A | ORF3b | ALB | 213 | 3 | albumin | ORFeome, fetal brain | -0,2 | 1,0 |
| A | NSP7 | YWHAE | 7531 | 2 | tyrosine 3-monooxygenase/tryptophan 5-monooxygenase activation protein, epsilon polypeptide | fetal brain | -0,3 | 1,2 |
| A | ORF7b | SERPING1 | 710 | 8 | serpin peptidase inhibitor, clade G (C1 inhibitor), member 1, (angioedema, hereditary) | fetal brain | -0,3 | 1,4 |
| A | NSP13 | MARK3 | 4140 | 4 | MAP/microtubule affinity-regulating kinase 3 | brain | -0,4 | 1,1 |
| **Category** | **Bait** | **Prey gene symbol** | **Prey gene id** | **Number of times this  prey has been isolated.** | **Description** | **Library for Y2H screening** | **LUMIER z-scores** | **signal to background ratios** |
| A | NSP9 | ENO1 | 2023 | 3 | enolase 1, (alpha) | brain, fetal brain | -0,6 | 0,8 |
| A | NSP13 | MARK2 | 2011 | 4 | MAP/microtubule affinity-regulating kinase 2 | brain | -0,6 | 1,2 |
| B | NSP3 | FAM108A1 | 81926 | 1 | family with sequence similarity 108, member A1 | fetal brain | 2,5 | 12,6 |
| B | NSP12 | PFDN5 | 5204 | 1 | prefoldin subunit 5 | brain | 2,0 | 4,4 |
| B | ORF6 | DCTN2 | 10540 | 1 | dynactin 2 (p50) | fetal brain | 1,8 | 5,1 |
| B | NSP7 | MIF4GD | 57409 | 1 | MIF4G domain containing | fetal brain | 1,7 | 7,7 |
| B | NSP12 | NDUFA10 | 4705 | 1 | NADH dehydrogenase (ubiquinone) 1 alpha subcomplex, 10, 42kDa | brain | 1,7 | 4,4 |
| B | NSP9 | C1orf142 | 116841 | 1 | chromosome 1 open reading frame 142 | fetal brain | 1,7 | 6,1 |
| B | NSP2 | SERPING1 | 710 | 1 | serpin peptidase inhibitor, clade G (C1 inhibitor), member 1, (angioedema, hereditary) | brain | 1,5 | 10,7 |
| B | ORF7a | VKORC1 | 79001 | 1 | vitamin K epoxide reductase complex, subunit 1 | fetal brain | 1,5 | 4,1 |
| B | NSP1 | LAS1L | 81887 | 1 | LAS1-like (S. cerevisiae) | fetal brain | 1,4 | 5,9 |
| B | ORF14 | DCTN2 | 10540 | 1 | dynactin 2 (p50) | brain | 1,4 | 3,8 |
| B | NSP5 | FKBP1A | 2280 | 1 | FK506 binding protein 1A, 12kDa | fetal brain | 1,2 | 5,2 |
| B | NSP8 | H2AFY2 | 55506 | 1 | H2A histone family, member Y2 | brain | 1,2 | 7,6 |
| B | ORF9b | RPS20 | 6224 | 1 | ribosomal protein S20 | fetal brain | 1,2 | 3,2 |
| B | NSP13 | CHEK2 | 11200 | 1 | CHK2 checkpoint homolog (S. pombe) | fetal brain | 1,1 | 2,5 |
| B | NSP8 | TERF1 | 7013 | 1 | telomeric repeat binding factor (NIMA-interacting) 1 | fetal brain | 1,1 | 2,6 |
| B | NSP6 | ISLR | 3671 | 1 | immunoglobulin superfamily containing leucine-rich repeat | brain | 0,8 | 3,0 |
| B | NSP12 | TBCB | 1155 | 1 | tubulin folding cofactor B | fetal brain | 0,8 | 2,9 |
| B | NSP4 | NMB | 4828 | 1 | neuromedin B | ORFeome | 0,7 | 1,7 |
| B | NSP4 | LAS1L | 81887 | 1 | LAS1-like (S. cerevisiae) | fetal brain | 0,7 | 2,3 |
| B | ORF3a | DCTN2 | 10540 | 1 | dynactin 2 (p50) | ORFeome | 0,6 | 2,2 |
| B | NSP12 | MNAT1 | 4331 | 1 | menage a trois homolog 1, cyclin H assembly factor (Xenopus laevis) | brain | 0,6 | 2,1 |
| B | NSP7 | NAE1 | 8883 | 1 | amyloid beta precursor protein binding protein 1 | fetal brain | 0,5 | 2,4 |
| B | NSP12 | HOXC6 | 3223 | 1 | homeobox C6 | ORFeome | 0,5 | 2,0 |
| B | ORF3a | YWHAE | 7531 | 1 | tyrosine 3-monooxygenase/tryptophan 5-monooxygenase activation protein, epsilon polypeptide | fetal brain | 0,5 | 2,8 |
| B | NSP8 | SERPING1 | 710 | 1 | serpin peptidase inhibitor, clade G (C1 inhibitor), member 1, (angioedema, hereditary) | fetal brain | 0,4 | 1,9 |
| B | NSP13 | SERPING1 | 710 | 1 | serpin peptidase inhibitor, clade G (C1 inhibitor), member 1, (angioedema, hereditary) | fetal brain | 0,4 | 2,6 |
| B | NSP7 | PLEKHO1 | 51177 | 1 | pleckstrin homology domain containing, family O member 1 | fetal brain | 0,3 | 1,9 |
| B | NSP15 | PPIA | 5478 | 1 | peptidylprolyl isomerase A (cyclophilin A) | fetal brain | 0,3 | 2,1 |
| B | NSP5 | N4BP2L2 | 10443 | 1 | phosphonoformate immuno-associated protein 5 | brain | 0,2 | 1,3 |
| B | NSP5 | XPA | 7507 | 1 | xeroderma pigmentosum, complementation group A | fetal brain | 0,2 | 2,1 |
| B | NSP8 | EIF4B | 1975 | 1 | eukaryotic translation initiation factor 4B | fetal brain | 0,1 | 2,3 |
| B | NSP5 | C20orf27 | 54976 | 1 | chromosome 20 open reading frame 27 | fetal brain | 0,1 | 1,4 |
| B | NSP7 | LCP1 | 3936 | 1 | lymphocyte cytosolic protein 1 (L-plastin) | brain | 0,1 | 1,7 |
| B | ORF7a | SMOC1 | 64093 | 1 | SPARC related modular calcium binding 1 | fetal brain | 0,0 | 1,4 |
| B | ORF3a | PFDN5 | 5204 | 1 | prefoldin subunit 5 | brain | 0,0 | 1,6 |
| B | ORF9b | RPS17 | 6218 | 1 | ribosomal protein S17 | ORFeome | 0,0 | 1,8 |
| B | NSP2 | NPHP3 | 27031 | 1 | nephronophthisis 3 (adolescent) | brain | -0,1 | 1,2 |
| B | ORF6 | BRF1 | 2972 | 1 | BRF1 homolog, subunit of RNA polymerase III transcription initiation factor IIIB (S. cerevisiae) | fetal brain | -0,2 | 1,2 |
| B | ORF14 | C7orf36 | 57002 | 1 | chromosome 7 open reading frame 36 | fetal brain | -0,3 | 1,2 |
| B | NSP13 | OCIAD2 | 132299 | 1 | OCIA domain containing 2 | brain | -0,3 | 1,0 |
| B | NSP2 | SLC46A3 | 283537 | 1 | solute carrier family 46, member 3 | brain | -0,5 | 1,1 |
| B | NSP13 | N4BP2L2 | 10443 | 1 | phosphonoformate immuno-associated protein 5 | brain | -0,5 | 0,7 |
