# Supplementary material for: The SARS-Coronavirus-Host Interactome: Identification of Cyclophilins as Target for Pan-Coronavirus Inhibitors
Source: PLoS Pathog. 2011 Oct 27;7(10):e1002331. doi: 10.1371/journal.ppat.1002331 (PMC3203193; doi:10.1371/journal.ppat.1002331)
Supplement: Table S2 — Identification of previously published SARS-CoV interactions with cellular proteins. Literature interactions were identified using a combination of text mining and manual curation. Abstracts on SARS containing a human protein and a mentioning of experimental methods such as yeast two-hybrid, Co-Immunoprecipitation or GST pulldown assay were manually screened for interactions between a human and a SARS protein. In the same way, human proteins enriched in SARS abstracts were investigated for interactions. In this way, 28 known interactions between SARS proteins and their human interaction partners were identified. “Y2H this study” (last column) hits refer to human genes identified here and in the literature. (DOC) [file ppat.1002331.s005.doc]

| **SARS** | **SARS protein** | **Human Prot-** | **Human protein** | **Human Protein** | **Reference** | **Y2H: this** |
| --- | --- | --- | --- | --- | --- | --- |
| **protein** | **(name in abstract)** | **ein (Symbo)** | **(Entrez)** | **(name in** | **(PMID)** | **study** |
|  |  |  | Gene) | abstract) |  |  |
| Nsp13 | nsp13 | DDX5 | 1655 | Ddx5 | 19224332 |  |
| Nsp10 | nsp10 | C11orf74 | 119710 | HEPIS | 18433331 |  |
| S | spike (S) protein | EIF3F | 8665 | eIF3f | 18231581 |  |
| N | N protein | EEF1A1 | 1915 | EF1alpha | 18448518 | lowfi |
| X1/Orf3a | Orf3a/X1/U274 | CAV1 | 857 | caveolin-1 | 17947532 |  |
| M | membrane (M) protein | IKBKB | 3551 | IKKbeta | 17705188 |  |
| N | N protein | UBE2I | 7329 | Ubc9 | 17037517 |  |
| X4/Orf7a | SARS-CoV 7a | SGTA | 6449 | hSGT | 16580632 | lowfi |
|  | (prev desig U122 and X4) | |  |  |  |  |
| Nsp5 | SARS-CoV 3CL(pro) protease | ATP6V1G1 | 9550 | vacuolar-H+ ATPase G1 subunit | 16226257 | lowfi |
| Nsp10 | nsp10 | BTF3 | 689 | BTF3 | 16157265 |  |
| Nsp10 | nsp10 | ATF5 | 22809 | ATF5 | 16157265 |  |
| Nsp10 | nsp10 | ND4L | 4539 | NADH-4L | 16157265 |  |
| Nsp10 | nsp10 | COX2 | 4513 | Cyto-II | 16157265 |  |
| N | nucleocapsid protein of SARS_CoV (SARS_N) | HNRPA1 | 3178 | hnRNP A1 | 15862300 |  |
| S | SARS-CoV spike (S)-protein | ACE2 | 59272 | ACE2 | 15791205 |  |
| S | spike protein | CLEC4G | 339390 | LSECtin | 16051304 |  |
| S | SARS-CoV S | CD209 | 30835 | DC-SIGN | 15479853 |  |
| S | SARS-CoV S | CLEC4M | 10332 | DC-SIGNR | 15479853 |  |
| Nsp3 | papain-like protease (PLpro) | IRF3 | 3661 | IRF-3 | 17761676 |  |
| Orf6 | ORF6 | KPNA2 | 3838 | karyopherin alpha 2 | 17596301 |  |
| S | spike-protein (S-protein) | SFTPD | 6441 | lung surfactant protein D (SP-D) | 17412287 |  |
| N | Nucleocapsid (N) protein | PPIA | 5478 | PPIA | 15688292 | hifi+lowfi |
| X4/Orf7a | SARS-CoV 7a protein | BCL2L1 | 598 | bcl-xL | 17428862 |  |
| X4/Orf7a | SARS-CoV 7a protein | BCL2L2 | 599 | Bcl-w | 17428862 |  |
| X4/Orf7a | SARS-CoV 7a protein | MCL1 | 4170 | Mcl-1 | 17428862 |  |
| X4/Orf7a | SARS-CoV 7a protein | BCL2A1 | 597 | A1 | 17428862 |  |
| X4/Orf7a | SARS-CoV 7a protein | BCL2 | 596 | Bcl-2 | 17428862 |  |
| E | small envelope E protein | BCL2L1 | 598 | Bcl-xL | 16048439 |  |
