# Supplementary material for: The SARS-Coronavirus-Host Interactome: Identification of Cyclophilins as Target for Pan-Coronavirus Inhibitors
Source: PLoS Pathog. 2011 Oct 27;7(10):e1002331. doi: 10.1371/journal.ppat.1002331 (PMC3203193; doi:10.1371/journal.ppat.1002331)
Supplement: Table S3 — Screening of more than 5,000 abstracts with a human synonym protein list (31,941 entries) on SARS coronavirus using the Text-Mining program syngrep for the occurrence of human targets of SARS proteins. Interaction partners of SARS-CoV identified in this study are enriched for proteins associated with SARS infection in previous studies. Numbers mean that e.g. in the case of 100 human protein synonyms six SARS or coronavirus protein entries were found. (DOC) [file ppat.1002331.s006.doc]

| **Data set** | **Fraction of SARS-CoV interaction partners found by HTY2H (contained in human protein synonym list)** | **Fraction of all proteins found** | **P-Value** |
| --- | --- | --- | --- |
| SARS interaction partners (hifi) | 6/100 =6% | 884 31,941 =2.8% | 0.0594 |
| SARS interaction partners (complete) | 36/514=7% | 884/31,941=2.8% | 4.4e-07 |
