# Supplementary material for: The SARS-Coronavirus-Host Interactome: Identification of Cyclophilins as Target for Pan-Coronavirus Inhibitors
Source: PLoS Pathog. 2011 Oct 27;7(10):e1002331. doi: 10.1371/journal.ppat.1002331 (PMC3203193; doi:10.1371/journal.ppat.1002331)
Supplement: Table S4 — Gene Ontology over-representation analysis performed on high confidence nsp1-targets. Among them were five proteins displaying peptidyl-prolyl cis-trans isomerase activity. BH = Multiple testing correction with Benjamine-Hochberg. P value cutoff was 0.05. (DOC) [file ppat.1002331.s007.doc]

| GO ID | Name | P-Value (BH) | SARS Targets |
| --- | --- | --- | --- |
| GO:0003755 | peptidyl-prolyl cis-trans isomerase activity | 8.61E-07 | PPIH, PPIG, PPIA, FKBP1A, FKBP1B |
| GO:0016859 | cis-trans isomerase activity | 8.61E-07 | PPIH, PPIG, PPIA, FKBP1A, FKBP1B |
| GO:0006457 | protein folding | 3.81E-05 | ST13, PPIH, PPIG, PPIA, FKBP1A, FKBP1B |
| GO:0043666 | regulation of phosphoprotein phosphatase activity | 1.46E-04 | RCAN1, FKBP1A, FKBP1B |
| GO:0016853 | isomerase activity | 2.37E-04 | PPIH, PPIG, PPIA, FKBP1A, FKBP1B |
| GO:0032512 | regulation of protein phosphatase type 2B activity | 2.38E-04 | FKBP1A, FKBP1B |
| GO:0032513 | negative regulation of protein phosphatase type 2B activity | 2.38E-04 | FKBP1A, FKBP1B |
| GO:0008144 | drug binding | 2.38E-04 | PPIH, PPIG, FKBP1A, FKBP1B |
| GO:0010921 | regulation of phosphatase activity | 2.39E-04 | RCAN1, FKBP1A, FKBP1B |
| GO:0035303 | regulation of dephosphorylation | 8.54E-04 | RCAN1, FKBP1A, FKBP1B |
| GO:0032515 | negative regulation of phosphoprotein phosphatase activity | 0.001027416 | FKBP1A, FKBP1B |
| GO:0010923 | negative regulation of phosphatase activity | 0.001176135 | FKBP1A, FKBP1B |
| GO:0034765 | regulation of transmembrane ion transport | 0.001176135 | FKBP1A, PDZK1, FKBP1B |
| GO:0005219 | ryanodine-sensitive calcium-release channel activity | 0.001176135 | FKBP1A, FKBP1B |
| GO:0005218 | intracellular ligand-gated calcium channel activity | 0.001176135 | FKBP1A, FKBP1B |
| GO:0022417 | protein maturation via protein folding | 0.001176135 | FKBP1A, FKBP1B |
| GO:0034762 | regulation of transmembrane transport | 0.00128052 | FKBP1A, PDZK1, FKBP1B |
| GO:0016018 | cyclosporin A binding | 0.002191307 | PPIH, PPIG |
| GO:0060314 | regulation of ryanodine-sensitive calcium-release channel activity | 0.002501986 | FKBP1A, FKBP1B |
| GO:0005528 | FK506 binding | 0.002501986 | FKBP1A, FKBP1B |
| GO:0005527 | macrolide binding | 0.002501986 | FKBP1A, FKBP1B |
| GO:0015278 | calcium-release channel activity | 0.00306772 | FKBP1A, FKBP1B |
| GO:0006458 | 'de novo' protein folding | 0.004288119 | FKBP1A, FKBP1B |
| GO:0042026 | protein refolding | 0.004288119 | FKBP1A, FKBP1B |
| GO:0016311 | dephosphorylation | 0.004900992 | PTPN2, RCAN1, FKBP1A, FKBP1B |
| GO:0034220 | transmembrane ion transport | 0.008105291 | FKBP1A, PDZK1, FKBP1B |
| GO:0005217 | intracellular ligand-gated ion channel activity | 0.008277177 | FKBP1A, FKBP1B |
| GO:0051004 | regulation of lipoprotein lipase activity | 0.012589845 | FKBP1A, FKBP1B |
| GO:0043269 | regulation of ion transport | 0.014729329 | FKBP1A, PDZK1, FKBP1B |
| GO:0032412 | regulation of ion transmembrane transporter activity | 0.028460934 | FKBP1A, FKBP1B |
| GO:0005488 | binding | 0.034013109 | 25 Genes |
| GO:0022898 | regulation of transmembrane transporter activity | 0.034013109 | FKBP1A, FKBP1B |
| GO:0034764 | positive regulation of transmembrane transport | 0.034556429 | PDZK1 |
| GO:0034767 | positive regulation of transmembrane ion transport | 0.034556429 | PDZK1 |
| GO:0021528 | commissural neuron differentiation in the spinal cord | 0.034556429 | C1orf187 |
| GO:0005124 | scavenger receptor binding | 0.034556429 | PDZK1 |
| GO:0034205 | beta-amyloid formation | 0.034556429 | FKBP1A |
| GO:0032409 | regulation of transporter activity | 0.041174979 | FKBP1A, FKBP1B |
| GO:0016529 | sarcoplasmic reticulum | 0.042036616 | FKBP1A, FKBP1B |
| GO:0016528 | sarcoplasm | 0.042896963 | FKBP1A, FKBP1B |
