# Supplementary material for: The SARS-Coronavirus-Host Interactome: Identification of Cyclophilins as Target for Pan-Coronavirus Inhibitors
Source: PLoS Pathog. 2011 Oct 27;7(10):e1002331. doi: 10.1371/journal.ppat.1002331 (PMC3203193; doi:10.1371/journal.ppat.1002331)
Supplement: Table S5 — Protein complexes preferentially targeted by SARS proteins. Shown are the four significantly enriched SARS-CoV targeted protein complexes. (DOC) [file ppat.1002331.s008.doc]

| **SARS-CoV targeted protein complex** | **All subunits of complex** | **Subunits int-eracting with SARS-CoV** | **Number**  **of inter-**  **acting**  **subunits** | **P-Value**  **(BH)** |
| --- | --- | --- | --- | --- |
| ***Respiratory chain complex I*** | mt-nd1,  mt-nd2,  mt-nd3,  mt-nd4l,  mt-nd5,  mt-nd6, ndufa1, ndufa10, ndufa11, ndufa12, ndufa13, ndufa2, ndufa3, ndufa4, ndufa5, ndufa6, ndufa7, ndufa8, ndufa9, ndufab1, ndufb1, ndufb10, ndufb11, ndufb2, ndufb3, ndufb4, ndufb5, ndufb6, ndufb7, ndufb8, ndufb9, ndufc1, ndufc2, ndufs1, ndufs2, ndufs3, ndufs4, ndufs5, ndufs6, ndufs7, ndufs8, ndufv1, ndufv2, ndufv3 | ndufb3, ndufs6, ndufa10, ndufs4, ndufa2, ndufb7, ndufa4 | 7 | 0.04 |
| ***Cytoplasmic ribosome*** | fau, rpl10, rpl10a, rpl11, rpl12, rpl13, rpl13a, rpl14, rpl15, rpl17, rpl18, rpl18a, rpl19, rpl21, rpl22, rpl23, rpl23a, rpl24, rpl26, rpl27, rpl27a, rpl28, rpl29, rpl3, rpl30, rpl31, rpl32, rpl34, rpl35, rpl35a, rpl36, rpl36a, rpl37, rpl37a, rpl38, rpl39, rpl4, rpl41, rpl5, rpl6, rpl7, rpl7a, rpl8, rpl9, rplp0, rplp1, rplp2, rps10, rps11, rps12, rps13, rps14, rps15, rps15a, rps16, rps17, rps18, rps19, rps2, rps20, rps21, rps23, rps24, rps25, rps26, rps27, rps27a, rps28, rps29, rps3, rps3a, rps4x, rps5, rps6, rps7, rps8, rps9, rpsa, uba52 | rps17, rpl8, rplp0, rpl37a, rpl31, rpsa, rpl10, rpl21, rpl12, rps20 | 10 | 0.04 |
| ***60S ribosomal subunit*** | rpl10, rpl10a, rpl11, rpl12, rpl13, rpl13a, rpl14, rpl15, rpl17, rpl18, rpl18a, rpl19, rpl21, rpl22, rpl23, rpl23a, rpl24, rpl26, rpl27, rpl27a, rpl28, rpl29, rpl3, rpl30, rpl31, rpl32, rpl34, rpl35, rpl35a, rpl36, rpl36a, rpl37, rpl37a, rpl38, rpl39, rpl4, rpl41, rpl5, rpl6, rpl7, rpl7a, rpl8, rpl9, rplp0, rplp1, rplp2, uba52 | rpl8, rplp0, rpl37a, rpl31, rpl10, rpl21, rpl12 | 7 | 0.04 |
| ***LCR-associated remodeling complex*** | actb, actl6a, arid1a, chd4, dpf2, gatad2b, hdac1, hdac2, hnrnpc, mbd2, mbd3, mta2, rbbp4, smarca4, smarcb1, smarcc1, smarcc2, smarcd2, smarce1 | hdac2, smarcd2, smarcb1, actb | 4 | 0.04 |
