# Supplementary material for: The SARS-Coronavirus-Host Interactome: Identification of Cyclophilins as Target for Pan-Coronavirus Inhibitors
Source: PLoS Pathog. 2011 Oct 27;7(10):e1002331. doi: 10.1371/journal.ppat.1002331 (PMC3203193; doi:10.1371/journal.ppat.1002331)
Supplement: Table S6 — Protein complexes preferentially targeted by SARS proteins. Frequency of SARS targets within protein complexes was compared to the overall frequency of protein subunits and was found to be significantly enriched compared to the overall background. (DOC) [file ppat.1002331.s009.doc]

| **Dataset** | **Percentage of SARS targets in complexes** | **Percentage of proteins in complexes** | **P-value** |
| --- | --- | --- | --- |
| High-conf. set | 21/108=19.4% | 2,079/25,000=4.3% | 0.0002 |
| Complete set | 113/610=18.5% | 2,079/25,000=4.3% | 3.33e-16 |
